# Uncovering inherent cellular plasticity of multiciliated ependyma leading to ventricular wall transformation and hydrocephalus

**DOI:** 10.1038/s41467-018-03812-w

**Published:** 2018-04-25

**Authors:** Khadar Abdi, Chun-Hsiang Lai, Patricia Paez-Gonzalez, Mark Lay, Joon Pyun, Chay T. Kuo

**Affiliations:** 1 Department of Cell Biology, Duke University School of Medicine, Durham, NC 27710 USA; 2 Department of Neurobiology, Duke University School of Medicine, Durham, NC 27710 USA; 3 Preston Robert Tisch Brain Tumor Center, Duke University School of Medicine, Durham, NC 27710 USA; 4 Brumley Neonatal/Perinatal Research Institute, Duke University School of Medicine, Durham, NC 27710 USA; 5 Institute for Brain Sciences, Duke University School of Medicine, Durham, NC 27710 USA

## Abstract

Specialized, differentiated cells often perform unique tasks that require them to maintain a stable phenotype. Multiciliated ependymal cells (ECs) are unique glial cells lining the brain ventricles, important for cerebral spinal fluid circulation. While functional ECs are needed to prevent hydrocephalus, they have also been reported to generate new neurons: whether ECs represent a stable cellular population remains unclear. Via a chemical screen we found that mature ECs are inherently plastic, with their multiciliated state needing constant maintenance by the Foxj1 transcription factor, which paradoxically is rapidly turned over by the ubiquitin-proteasome system leading to cellular de-differentiation. Mechanistic analyses revealed a novel NF-κB-independent IKK2 activity stabilizing Foxj1 in mature ECs, and we found that known IKK2 inhibitors including viruses and growth factors robustly induced Foxj1 degradation, EC de-differentiation, and hydrocephalus. Although mature ECs upon de-differentiation can divide and regenerate multiciliated ECs, we did not detect evidence supporting EC’s neurogenic potential.

## Abstract (web summary)

Multiciliated ependymal cells (ECs) in the mammalian brain are glial cells facilitating cerebral spinal fluid movement. This study describes an inherent cellular plasticity of ECs as maintained by Foxj1 and IKK2 signaling, and shows resulting hydrocephalus when EC de-differentiation is triggered.

## Introduction

The adult mammalian brain has limited intrinsic capacity to regenerate. While there are resident neuronal and glial progenitors with restricted proliferative potentials, the vast majority of cell types in the adult brain are terminally differentiated. Different subpopulations of mature neurons and glia perform specific tasks, exhibit complex/distinct cellular morphologies, no longer self-renew or divide, and are generally considered the basal differentiated states for the cells. Multiciliated ependymal cells (ECs) are specialized epithelial cells lining the mammalian brain ventricles throughout, bathed by cerebral spinal fluid (CSF) along their apical surfaces^1^. ECs are derived from Nestin^+^ radial glial progenitors during their terminal differentiation in development^2–7^, temporally coinciding with a transition from embryonic to postnatal neurogenesis along the lateral brain ventricles^8^. During radial glial differentiation to a distinctive multiciliated phenotype, ECs acquire specialized interdigitating lateral membranes^4,9^, with planar polarity cues controlling multicilia beating orientations on their cellular surfaces^10,11^. Although ECs form perhaps the largest epithelial surface in the brain, their specific functions and contributions to central nervous system (CNS) physiology are poorly understood. Genetic mutant mouse models have shown that ependymal disruptions during development can result in hydrocephalus/ventriculomegaly^12,13^, as well as postnatal neurogenic niche assembly defects^4^. Hydrocephalus remains the most prevalent form of developmental CNS malformation, though interestingly inheritable genetic mutations in human hydrocephalus are relatively rare, while trauma, intraventricular hemorrhages, and CNS infections account for most cases^14^.

ECs were once believed to be the source of new neurons in the postnatal mammalian brain^15,16^. However, in recent years, this notion has been revised to an understanding that mature ECs retain the capacity to generate newborn neurons under certain environmental stimuli, such as brain injury^17^ or growth factor exposure^18^, but otherwise remain in a non-proliferative state. Since the adult mammalian CNS has limited capacity to regenerate after injury or degeneration, the notion that mature ependyma can serve as a neurogenic reservoir requires critical examination. At the heart of this long running debate on whether multiciliated ECs retain neurogenic capacity, is the phenotypic stability of a seemingly terminally differentiated multiciliated epithelial cell in the brain. However, this important and challenging problem has not been directly addressed.

The transcription factor Foxj1 belongs to the large forkhead domain DNA-binding protein family^19^. It is a critical regulator of motile-ciliated cellular development, including the primitive node^20^, multiciliated cells lining the trachea^21^, and the ependyma^3,4^. We showed previously that *foxj1* expression is induced during terminal maturation of ventricular radial glial progenitors specified to becoming ECs^4^. And *foxj1* deletion resulted in radial glial differentiation arrest and the complete lack of multiciliated ECs^3,4^. Performing a chemical screen for modulators of EC differentiation, we discovered unexpectedly that the mature multiciliated ependymal phenotype is not fixed, needing constant Foxj1 protein expression to prevent cellular de-differentiation back to a glial-like morphology. Paradoxically, we found that ependymal Foxj1 protein has a short half-life, requiring non-canonical IκB kinase (IKK) activity to prevent rapid degradation via the ubiquitin proteasome system (UPS). Armed with these novel molecular insights, we demonstrated that this previous unknown signaling pathway is directly targeted by serum and viruses to induce EC transformation, ventricular breakdown, and hydrocephalus. While de-differentiated mature ECs can divide and regenerate ECs, we did not find evidence to support their neurogenic capacity. We believe these findings provide a foundational basis for EC biology/brain ventricle stability research in health and disease moving forward.

## Results

### Chemical screen for ependymal differentiation modulators

**Fig. 1:**
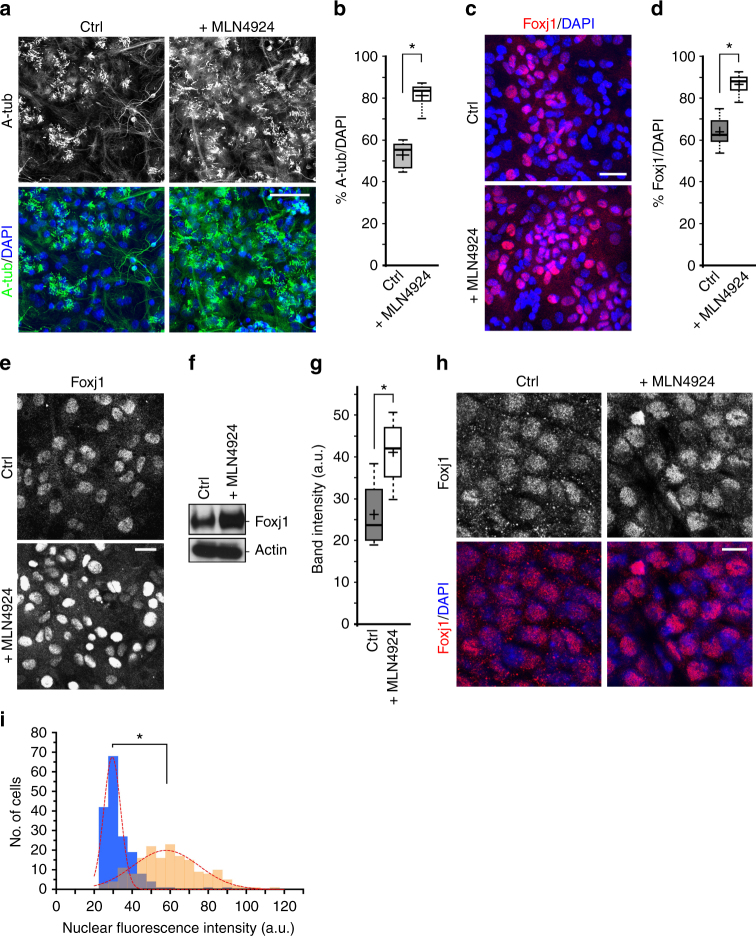
Effects of MLN4924 on ependymal Foxj1 transcription factor expression. **a** Representative IHC staining of control (Ctrl) and MLN4924-treated primary EC cultures, labeled with acetylated tubulin (A-tub) antibody and DAPI. Scale bar: 50 µm. **b** Quantification of multiciliated cell numbers as a fraction of DAPI^+^ nuclei. *P < 0.008, Wilcoxon two-sample test, *n* = 5, *z* = 1.107. **c** Representative IHC staining from Ctrl and MLN4924-treated primary EC cultures, labeled with Foxj1 antibody and DAPI. Scale bar: 30 µm. **d** Quantification of number of Foxj1^+^ cells as a fraction of DAPI nuclei in untreated and MLN4924-treated primary EC cultures. *P < 0.008, Wilcoxon two-sample test, *n* = 5, *z* = 1.331. **e** IHC staining of differentiated primary EC cultures without (Ctrl) or with MLN4924 treatment for 12 h, labeled with Foxj1 antibody and DAPI. Note the increase in relative fluorescence intensity for Foxj1 in MLN4924-treated cultures. Scale bar: 20 µm. **f** Western blot analysis showing relative Foxj1 protein levels from primary EC cultures without (Ctrl) or with MLN4924 treatment for 12 h. Actin is used as protein loading control. **g** Quantification of relative amounts of Foxj1 protein from western blot analyses in **f**. *P < 0.03, Wilcoxon two-sample test, *n* = 4, *z* = 1.316. **h** IHC staining of fixed ependymal wholemounts from P28 animal without (Ctrl) or with MLN4924 treatment for 6 h, labeled with Foxj1 and DAPI. Scale bar: 10 µm. **i** Quantification of cell numbers with indicated Foxj1 IHC fluorescence intensities, presented as histogram comparing untreated (blue) vs. 24 h MLN4924 treatment (orange). Dashed lines represent Gaussian fit distribution curve. *P < 0.0001, unpaired Student’s *t* test, *n* = 172 in each group, *t*_171_ = 49.780. Box plots show mean (+), median (−), quartiles (boxes), range (whiskers)

To identify molecular pathways that can modulate multiciliated EC differentiation from postnatal radial glial progenitors (pRGPs), we utilized an in vitro ependymal assay that we had previously developed^4^. Culturing primary progenitors from the lateral ventricular surface of newborn mice, we observed that roughly 52.4 ± 6.4% of these cells turned into multiciliated ECs during in vitro differentiation (Fig. 1a, b, Supplementary Fig. 1a). Consistent with in vivo markers of multiciliated EC differentiation^5,6^, p73 and c-Myb were also expressed during early ependymal culture differentiation and downregulated in mature ECs (Supplementary Fig. 1b). We reasoned that by altering culturing media composition and scoring percentages of multiciliated cells, this primary culture assay presented an excellent platform to screen for novel pathways regulating production/maintenance of mature ECs. Thus we initiated a chemical screen for potential small molecules that could further enrich multiciliated EC production during in vitro differentiation.

We have identified one compound MLN4924 that can consistently increase EC numbers in vitro. MLN4924 treatment during ependymal progenitor differentiation resulted in 81.1 ± 6.5% of cells in primary pRGP culture becoming multiciliated as assessed by acetylated-tubulin + DAPI immunohistochemistry (IHC) staining (Fig. 1a, b, Supplementary Fig. 1a). We also used Foxj1 transcription factor expression as a readout for the presence of ECs^4^, as multiciliated ECs in culture uniformly showed Foxj1 protein by IHC staining (Supplementary Fig. 1c). Consistent with the observed increase in multiciliated cells in culture, Foxj1 + DAPI IHC staining showed similarly robust increase in co-localization percentage in MLN4924-treated vs. control conditions (Fig. 1c, d, Supplementary Fig. 1d). IHC staining revealed increased Foxj1 fluorescence intensity in many cells compared to untreated controls (Fig. 1c), suggesting that MLN treatment during ependymal differentiation increased both total numbers of Foxj1^+^ cells, as well as Foxj1 protein content per cell. Interestingly, in fully differentiated ependymal cultures without prior MLN4924 treatment, acute MLN4924 addition for 12 h resulted in consistently higher overall level of Foxj1 IHC staining compared to untreated controls (Fig. 1e), further supported by western blot analyses (Fig. 1f, g, Supplementary Fig. 13). In freshly isolated mature ECs from P28 brain ventricular wall tissue (wholemount preparation), acute MLN4924 treatment also resulted in notable increase in Foxj1 IHC fluorescence level vs. untreated control (Fig. 1h). Activated Caspase-3 IHC staining showed no obvious differences between control and MLN4924-treated EC cultures during differentiation, and quantification of Foxj1 IHC staining fluorescence intensity in individual ECs showed a robust population increase in Foxj1 protein levels (Fig. 1i).

### Inherent Foxj1 protein instability in mature ependymal cells

**Fig. 2:**
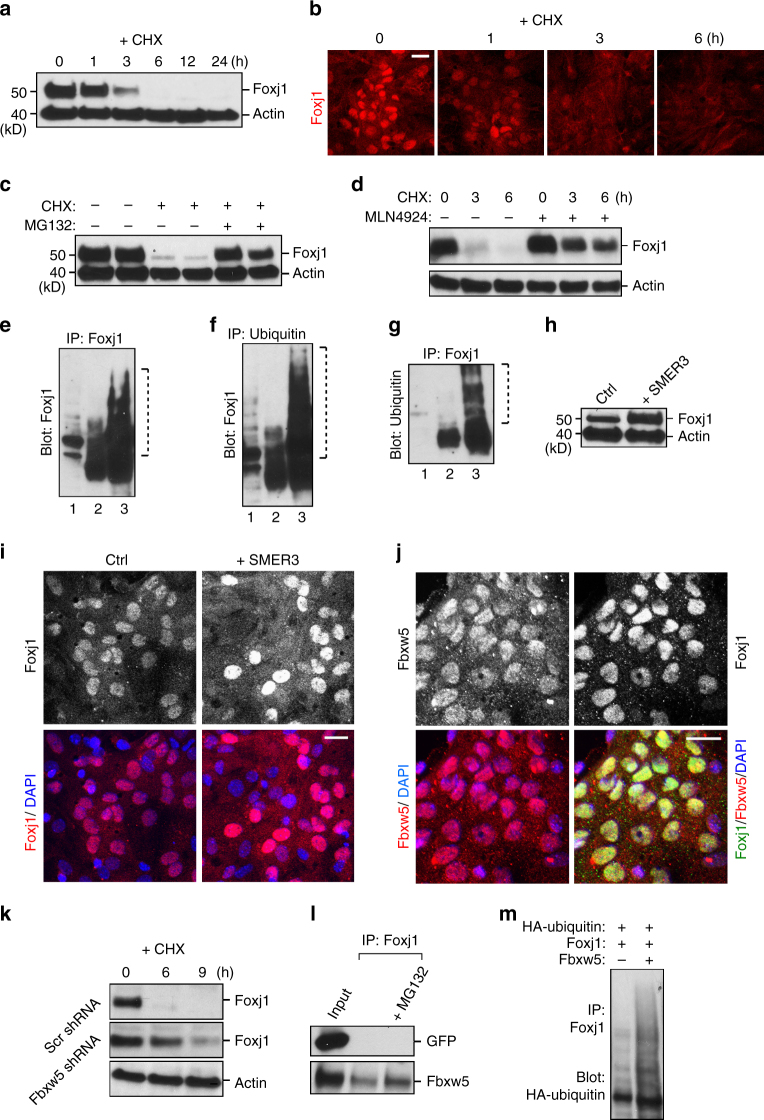
Ependymal Foxj1 protein instability and turnover. **a**, **c**,** d**,** h**, **k** Western blot (WB) analyses of protein lysates from primary EC cultures. **a** Foxj1 protein levels before and after treatment with cycloheximide (CHX) for indicated lengths of time. Actin = loading control. **b** IHC staining of EC cultures treated with CHX for the indicated lengths of time, labeled with Foxj1 antibody. Foxj1 is lost at a similar timescale as observed in WBs. **c** Foxj1 protein levels in untreated controls, CHX-treated for 3 h, or co-treated with CHX + MG132 for 3 h. Actin = loading control. **d** Foxj1 protein levels in untreated controls, CHX-treated, or co-treated with CHX + MLN4924 for the indicated lengths of time. Actin = loading control. **e** Immunoprecipitation (IP) of Foxj1 from EC cultures followed by WB probing for Foxj1 to reveal ubiquitin-induced laddering of Foxj1 protein. Lane 1 = input, lane 2 = IP Foxj1, lane 3 = IP of Foxj1 after MG132 treatment for 6 h. **f** IP of ubiquitin from EC cultures followed by WB probing for Foxj1. Lane 1 = input, lane 2 = IP ubiquitin, lane 3 = IP ubiquitin following MG132 treatment for 6 h. **g** IP of Foxj1 from EC cultures followed by WB probing for ubiquitin. Lane 1 = input, lane 2 = IP of Foxj1, lane 3 = IP of Foxj1 following MG132 treatment for 6 h. **h** Cultures treated with DMSO control (Ctrl) or SMER3, probed for Foxj1 and Actin. **i** Representative IHC staining of primary EC cultures without (Ctrl) or with SMER3 treatment, labeled with Foxj1 antibody and DAPI. Note the increased Foxj1 IHC signal in SMER3-treated EC cultures. **j** IHC staining of differentiated EC cultures labeled with Fbxw5, Foxj1 antibodies, and DAPI. **k** Cultures harboring scrambled control (Scr) shRNA or Fbxw5 shRNA, treated with CHX for indicated lengths of time, and probed for Foxj1 and Actin. **l** IP of Foxj1 from HEK293 cells either co-transfected with GFP and Foxj1-Myc expression constructs, or Fbxw5 and Foxj1-Myc constructs. WBs were probed with GFP or Fbxw5 antibodies. Lane 1 = inputs, Lane 2 = IP of Foxj1, Lane 3 = IP of Foxj1 following 6 h MG132 treatment. **m** IP of Foxj1 from HEK293 cell lysates following co-transfection with expression constructs as indicated (+), probed with anti-HA antibody to detect HA-ubiquitin. Scale bars: 20 µm

MLN4924 is a neddylation inhibitor that specifically blocks cullin-RING family E3 ubiquitin ligases^22^, currently under human clinical trials for cancer therapy^23^. Since MLN4924 increased Foxj1 antibody immunofluorescence in ECs (Fig. 1e, g, h), and concurrently we did not detect obvious changes to Foxj1 mRNA levels following MLN4924 treatment (Supplementary Fig. 2a), we wondered whether MLN4924 may be blocking Foxj1 protein turnover. The baseline stability of ependymal Foxj1 protein is unknown, so to test this, we first treated differentiated primary ependymal cultures with cycloheximide (CHX) to block new protein synthesis. This resulted in rapid disappearance of Foxj1 protein at 3 h, complete by 6 h after treatment onset (Fig. 2a, b, Supplementary Fig. 13). As controls, CHX-mediated degradation of Cyclin D1 and βIII-tubulin proteins in ependymal cultures occurred at similar rates to those previously reported^24,25^ (Supplementary Fig. 2b). Proteasomal blocker MG132 (Fig. 2c, Supplementary Fig. 13), as well as MLN4924 (Fig. 2d, Supplementary Fig. 13), stabilized Foxj1 in the presence of CHX, implicating ubiquitin proteasome-mediated protein turnover. Immunoprecipitating (IP) Foxj1 from differentiated primary ependymal cultures and western blotting for Foxj1, we noticed increased laddering of Foxj1 in the presence of MG132 (Fig. 2e, Supplementary Fig. 13). We next performed IP experiments in ependymal cultures using (1) anti-ubiquitin antibody for IP then blotting with anti-Foxj1 antibody (Fig. 2f, Supplementary Fig. 13), and in reverse (2) IP-ing with anti-Foxj1 antibody then blotting with anti-ubiquitin antibody (Fig. 2g, Supplementary Fig. 13). They also showed consistent laddering of Foxj1 protein in the presence of MG132 after 6 h incubation (Fig. 2f, g, Supplementary Fig. 13). CHX treatment of acutely isolated ependymal wholemounts from P28 animals showed a similarly rapid disappearance of Foxj1 protein in mature ECs, which can be blocked by MLN4924 (Supplementary Fig. 2c). These results revealed that the Foxj1 transcription factor is rather unstable in the mature ependyma, constantly targeted for degradation by the UPS.

We next wanted to determine the molecular pathways regulating Foxj1 protein turnover. To uncover ubiquitin ligase(s) mediating Foxj1 degradation, we reasoned that a specific inhibitor of the relevant ligase should result in increased ependymal Foxj1 protein level. We screened known chemical blockers of ubiquitin ligases in differentiated primary ependymal culture and found that in addition to MLN4924, SMER3 was somewhat effective in increasing Foxj1 protein in ependymal cultures (Fig. 2h, i, Supplementary Fig. 13). SMER3 is a known inhibitor of yeast cullin-RING family ubiquitin ligase SCFMET30^26^. Based on sequence homology comparisons, the closest mammalian homologs of yeast SCFMET30 are cullin-RING ligase family members Fbxw5, Fbxw7, and Fbxw11^27–29^. IHC staining for Foxj1 protein localization in differentiated ependymal cultures during CHX treatment showed concentration inside the nucleus (Fig. 2b). To rule out the potential of a modified/cytoplasmic form of Foxj1 protein not recognized by the Foxj1 antibody, we utilized a previously generated Myc-tagged version of the Foxj1 protein^4^. Lentiviral-mediated Foxj1-Myc expression in primary ependymal cultures, now stained with anti-Myc instead of Foxj1 antibody, showed similar nuclear localization and turnover following CHX treatment (Supplementary Fig. 2d). Using antibodies specific to Fbxw family members, we found that Fbxw5 was localized in the nucleus of differentiated ECs in vitro and in vivo (Fig. 2j, Supplementary Fig. 2e). To assay the functions of Fbxw5 in Foxj1 protein turnover, we utilized shRNAs that can efficiently knockdown Fbxw5 expression (Supplementary Fig. 2f). Lentiviral-mediated shRNA knockdown of Fbxw5 in primary ependymal cultures specifically blunted CHX-induced Foxj1 degradation (Fig. 2k, Supplementary Fig. 13), indicating Fbxw5’s functional contribution to ubiquitin-proteasome-mediated Foxj1 turnover. Consistent with this notion, Foxj1 immunoprecipitated with Fbxw5 in primary ependymal cultures, enhanced by MG132-mediated proteasomal blockade (Fig. 2l, Supplementary Fig. 13). And Fbxw5 robustly enhanced Foxj1 ubiquitination level when co-transfected into HEK293 cells (Fig. 2m, Supplementary Fig. 13).

### Mature EC phenotype continues to require *foxj1* expression

**Fig. 3:**
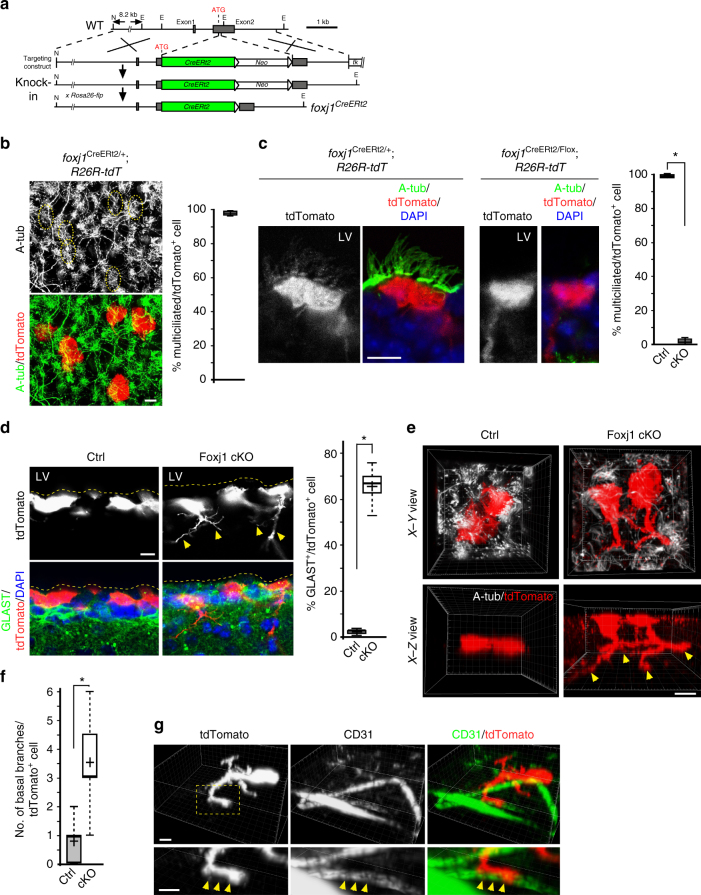
Inducible deletion of Foxj1 from mature ECs. **a** Schematic representation of *foxj1*^CreERt2/+^ gene targeting strategy, inserting CreER^t2^ into ATG site encoding Foxj1. N = NotI, E = EcoRI. Recombined allele was crossed to *Rosa26-flp*, removing neomycin selection cassette (*neo*) for proper CreER^t2^ recombinase expression from *foxj1* gene locus. **b**–**g** All experimental animals were tamoxifen induced at P14, samples harvested at P28. **b** Representative IHC staining of ependymal wholemount from *foxj1*^CreERt2/+^*; R26R-tdT* animal, labeled with acetylated tubulin (A-tub) and RFP antibodies. Dashed circles indicate multicilia bundles (upper panel) from individual tdTomato^+^ ECs. Quantification = % of tdTomato^+^ cells showing multicilia, from four animals. **c** Representative IHC staining of brain sections from *foxj1*^CreERt2/+^*; R26R-tdT* and *foxj1*^CreERt2/Flox^*; R26R-tdT* animals, labeled with A-tub and RFP antibodies, and DAPI, showing loss of multicilia in lineage-traced Foxj1 mutant tdTomato^+^ ECs. Quantification = % of tdTomato^+^ cells containing multicilia. *P < 0.008, Wilcoxon two-sample test, *n* = 5 mice, *z* = 1.583. **d** IHC images of brain sections from *foxj1*^CreERt2/+^*; R26R-tdT* and *foxj1*^CreERt2/Flox^*; R26R-tdT* animals, labeled with GLAST and RFP antibodies, and DAPI. Quantification = % of tdTomato^+^ ECs that are also GLAST^+^. *P < 0.008, Wilcoxon two-sample test, *n* = 5 mice, *z* = 1.228. **e** Imaris 3D rendering of tdTomato^+^ lineage-traced ECs from *foxj1*^CreERt2/+^*; R26R-tdT* and *foxj1*^CreERt2/Flox^*; R26R-tdT* animals, labeled with A-tub and RFP antibodies. *X*−*Y* (upper panels) and *X*−*Z* (lower panels) views illustrate morphological changes of Foxj1-mutant ECs extending basal processes (arrowheads). **f** Quantification of **e**: number of basal processes in tdTomato^+^ ECs from *foxj1*^CreERt2/+^*; R26R-tdT* (Ctrl) vs. *foxj1*^CreERt2/Flox^*; R26R-tdT* (cKO) animals, performed via Imaris 3D filament tracing. *P < 0.0001, unpaired Student’s *t *test; *n* = 20, *t*_18_ = 6.664. **g** Imaris 3D rendering of a representative tdTomato^+^ mutant EC from *foxj1*^CreERt2/Flox^*; R26R-tdT* animal, labeled with CD31 and RFP antibodies. Dashed box = close-up views shown in lower panels, revealing contact between basal process from mutant EC and CD31^+^ vasculature (arrowheads). Scale bars: 10 µm. Box plots show mean (+), median (−), quartiles (boxes), range (whiskers)

These findings are somewhat surprising, namely that the key transcription factor required for terminal differentiation of multiciliated ECs is itself unstable. To test whether sustained Foxj1 protein expression is required to maintain the mature cellular state after differentiation, we set out to inducibly delete Foxj1 from mature ECs. To visualize individual multiciliated ECs, we took a tamoxifen-inducible CreER genetic approach, combined with *Rosa26-tdTomato* (*R26R-tdT*) reporter mice, to generate tdTomato-labeled ECs. The 1 kb *FOXJ1* human promoter element has been used extensively over the past decade to label/target multiciliated cells in mice^30–33^, including brain ECs^3,4,17^. To exclude potential mis-labeling of non-EC types by the *FOXJ1-CreER*^*t2*^ transgenic driver^31^, we used homologous recombination to knock-in CreER^t2^ recombinase into the endogenous *foxj1* gene locus (Fig. 3a). While this fusion into the ATG start codon for *foxj1* disrupted the native gene, the resulting heterozygous *foxj1*^CreERt2/+^ mice were viable, fertile, and showed no obvious abnormalities, consistent with our previous observations in *foxj1*^KO/+^ heterozygous mice^4^. In *foxj1*^CreERt2/+^; *R26R-tdT* mice, tamoxifen induction in vivo at P14, analyzed 2 weeks later, showed specific tdTomato expression in multiciliated cells (Fig. 3b, c, Supplementary Fig. 3), confirming efficient and specific ependymal targeting by the *foxj1*^CreERt2/+^ driver.

We next crossed the *foxj1*^CreERt2/+^ driver to our previously generated *foxj1*^Flox/+^ conditional allele^4^ to produce *foxj1*^CreERt2/Flox^ mice. *R26R-tdT* was also crossed into the background to enable lineage-tracing experiments. These *foxj1*^CreERt2/Flox^ heterozygous mutant mice, similar to *foxj1*^KO/Flox^ mice that we generated previously^4^, showed no obvious phenotypic abnormalities in the absence of tamoxifen injection. In *foxj1*^CreERt2/Flox^; *R26R-tdT* conditional mutant mice (cKO), tamoxifen induction in vivo at P14, analyzed 2 weeks later showed highly effective removal of Foxj1 protein in tdTomato^+^ cells (Supplementary Fig. 4a). Upon *foxj1* inducible-deletion, these tdTomato^+^ mutant cells lost multicilia and their associated basal bodies (Fig. 3c), as well as aberrantly upregulated Glast^2,4^ and GFAP expression (Fig. 3d, Supplementary Fig. 4b, c). These results are in contrast to control *foxj1*^CreERt2/+^; *R26R-tdT* mice induced with the same tamoxifen schedule, showing tdTomato labeling in multiciliated Glast^−^, GFAP^−^, Foxj1^+^ ECs (Fig. 3c, d). More strikingly, while control ECs exhibited epithelial morphology, Foxj1 cKO mutant ECs extended complex basal cellular processes (Fig. 3d–f), terminating on CD31^+^ vascular endothelial cells (Fig. 3g), showing morphological features of subependymal glial progenitors^34^. Nissl staining of P28 Foxj1 cKO mutant brain sections, tamoxifen-induced at P14, showed clear ventriculomegaly/hydrocephalus (Supplementary Fig. 4d, e). Repeating these experiments via P30 tamoxifen induction, analyzed 2 weeks later, showed the same Foxj1 cKO mutant EC morphological changes, loss of multicilia and their associated basal bodies, as well as ventriculomegaly/hydrocephalus (Supplementary Fig. 5). The presence of Foxj1^+^ multiciliated ECs (not targeted by CreER) in tamoxifen-injected mutant mice showed that these phenotypic changes seen in Foxj1-deleted ECs was not induced by hydrocephalus (Fig. 3e, Supplementary Fig. 5a, e). These results demonstrated that continued Foxj1 protein expression is required for mature ECs to maintain their terminally differentiated multiciliated state, preventing hydrocephalus. They also showed that remarkably, the tissue stability of the ependyma is controlled by the turnover homeostasis of a single transcription factor.

### Non-canonical IKK2 control of EC Foxj1 protein stability

**Fig. 4:**
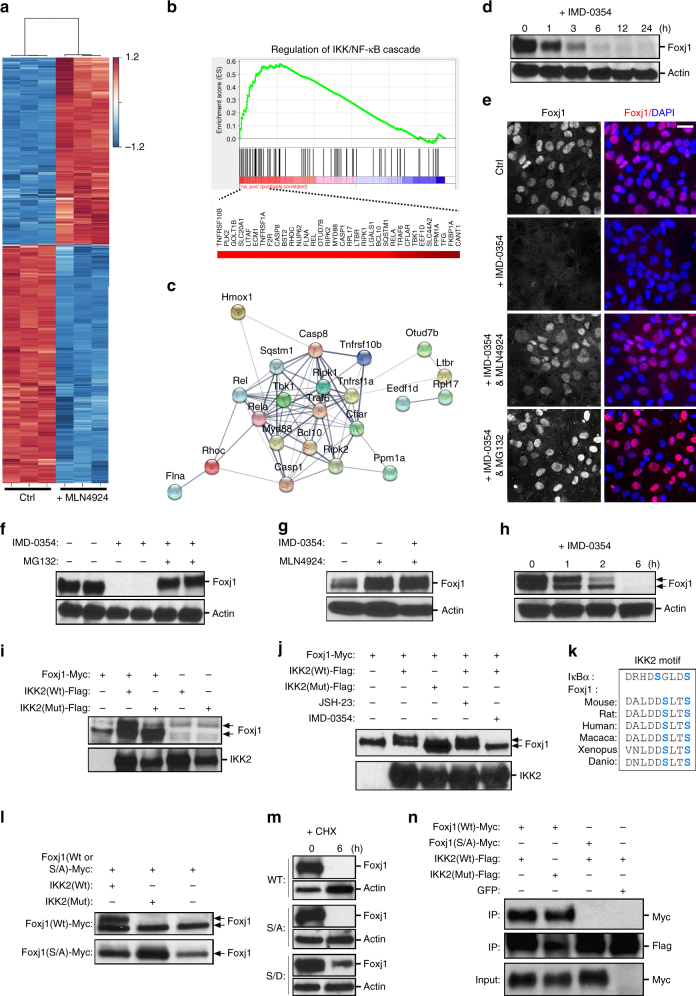
IKK function in Foxj1 stability. **a** Transcriptome heatmap (FDR ≤ 0.05) comparing EC cultures without (Ctrl) or with MLN4924 treatment, *Z*-score normalized. Samples/genes are clustered by correlation distance with complete linkage. **b** Gene enrichment plot of IKK/NF-κB cascade identified from gene set enrichment analysis (GSEA), using gene ontology (GO) database. Genes ranked on significance and direction of change (*t*-statistic). List of genes enriched within pathway (listed beneath) is presented with correlated heatmap varying in color from black to red. **c** Protein interaction network from identified genes within IKK/NF-κB cascade, using STRING functional protein association network. Connecting line thickness corresponds to network association confidence. **d**, **f**, **g**,** m** Western blot (WB) analyses of protein lysates from primary EC cultures. **d** Cultures treated with IMD-0354 for the indicated lengths of times, and probed for Foxj1, Actin (loading control). **e** Representative IHC staining of primary EC cultures without (Ctrl) or with addition of indicated compounds and combinations for 6 h, labeled with Foxj1 antibody and DAPI. Scale bar: 20 µm. **f** Cultures treated for 6 h with IMD-0354 alone or IMD-0354 + MG132, and probed for Foxj1, Actin. **g** Cultures untreated or treated for 6 h with MLN4924 or IMD-0354 + MLN4924, and probed for Foxj1, Actin. **h** Modified SDS-page gel protocol separating closely migrating molecular weight bands followed by WB for Foxj1, Actin. ECs were treated with IMD-0354 for indicated lengths of time. **i**, **j**, **l** Western blot (WB) analyses of protein lysates from HEK293 cells. **i** Cultures transfected with indicated expression constructs, and probed with anti-Foxj1 and anti-Flag (IKK2) antibodies. IKK2-Flag levels are used as expression controls. Note the formation of a Foxj1 doublet in the presence of IKK2(Wt) but not the IKK2(Mut) (kinase dead) conditions. **j** Cultures co-transfected with the indicated expression constructs and drug treatments, probed with anti-Foxj1, anti-Flag (IKK2) antibodies. IKK2-Flag levels = expression controls (lower lane). **k** IKK2 phosphorylation motif, comparing IκBα, Foxj1 proteins from several species. Alignment of target serines highlighted in blue. **l** Cultures co-transfected with expression constructs as indicated (+), probed with anti-Foxj1 antibody. **m** Cultures expressing indicated Foxj1 constructs, probed with anti-Myc antibody. Comparison of cultures untreated vs. treated with CHX for 6 h, showing enhanced stability of S32D/S35D (S/D)-Foxj1-Myc mutant protein (not S/A-Foxj1-Myc). **n** IP from HEK293 cell lysates co-transfected with expression constructs as indicated (+), probed with Myc(Foxj1) and Flag(IKK2) antibodies. IKK2-Flag and Foxj1-Myc levels are used as expression controls (lower lanes)

While MLN4924 can effectively block ependymal Foxj1 protein degradation, which biological pathways may accelerate this process? We reasoned that ependymal changes during MLN4924 treatment may reveal the relevant cellular machineries. We therefore performed transcriptome profiling, comparing MLN4924-treated vs. untreated primary ependymal cultures using expression microarrays. To examine molecular signatures, ependymal cultures were harvested for RNA 36 h following start of DMSO-only vs. MLN4924-treated conditions. Western blot analysis was performed concurrently to validate Foxj1 protein stabilization by MLN4924 treatment. Unsupervised hierarchical clustering clearly segregated transcriptional profiles based on treatment groups (Fig. 4a), suggesting a common cellular consequence in response to drug treatment. MLN4924 resulted in the upregulation of 899 genes and downregulation of 804 genes (>2-fold change, *P* < 0.001). Multiple genes in the dataset annotated as signal transduction regulators (Supplementary Fig. 6a), and the top five pathways implicated by gene ontology analysis included IKK/NF-κB signaling (Fig. 4b, c). MLN4924 is an effective inhibitor of cullin-RING E3 ligases that are important for cellular homeostasis in a variety of processes^22,35^; thus it was expected that “signal transduction regulation” would emerge as a top pathway in our analyses. In fact, there is extensive overlap in this gene cohort with those indicated in the IKK/NF-κB pathway (Fig. 4b, c, Supplementary Fig. 6a). While MLN4924 has been reported to alter IKK/NF-κB signaling in B-cell lymphoma cell line^36^, IKK/NF-κB functions in ECs as well as Foxj1 homeostasis are unknown.

The ependyma is in direct contact with the CSF, which is commonly used for diagnosing human CNS infections. The IKK/NF-κB pathway is a complex signaling hub, critically important in immune-mediated cellular responses^37^, thus we wondered whether it is involved in ependymal Foxj1 protein stability. We treated differentiated primary ependymal cultures with IKK-specific blockers, and found that remarkably, IKK2 inhibitor IMD-0354^38^ resulted in rapid degradation of Foxj1 protein (Fig. 4d, e, Supplementary Fig. 14), in a similar time course to CHX treatment (Fig. 2a, b, Supplementary Fig. 13). To determine whether IMD-0354 is inducing Foxj1 degradation through UPS function, we co-added IMD-0354 with MG132 or MLN4924, and either were effective in blocking IMD-0354-induced Foxj1 degradation (Fig. 4e–g, Supplementary Fig. 14). With MLN4924 co-incubation, IMD-0354 inhibition of IKK2 did not result in major changes to Foxj1 protein levels (Fig. 4g, Supplementary Fig. 14), suggesting that potential MLN4924 modulation of IKK2 activity is not a critical contributor to maintaining EC Foxj1 protein stability. We found similar effects of IMD-0354-induced Foxj1 degradation on freshly isolated mature ependymal wholemounts (Supplementary Fig. 6b), followed by loss of multicilia consistent with Foxj1’s transcriptional regulation of multiciliogenesis (Supplementary Fig. 6c). To rule out non-specific IMD-0354 toxicity, 3 days after IMD-0354 treatment washout, we observed re-expression of Foxj1 protein in primary ependymal cultures (Supplementary Fig. 6d). Moreover, shRNA knockdown of IKK2 in primary ependymal cultures resulted in robust decrease of Foxj1 protein levels: in multiple experiments we did not detect appreciable Foxj1 staining in GFP^+^ IKK2 shRNA-targeted cells (Supplementary Fig. 7a–c). These results suggested that constitutive NF-κB pathway activation is required to stabilize ependymal Foxj1 protein in the physiological state, but surprisingly, SC75741 and JSH-23, compounds that specifically block NF-κB transcription factor activity did not result in ependymal Foxj1 degradation (Supplementary Fig. 7d).

The IKK complex is best known for its important role in NF-κB pathway activation^39^, primarily through subunit IKK2 kinase activity^40,41^, although NF-κB-independent/non-canonical IKK functions have also been described^42^. To determine whether IKK’s role in Foxj1 stability may be through direct protein phosphorylation independent of NF-κB activation, we first treated primary ependymal cultures with IMD-0354 for 1 and 2 h and performed optimized SDS-page protocol to detect molecular weight bands for Foxj1 protein. This resulted in clear distinction of two close Foxj1 bands (Fig. 4h, Supplementary Fig. 14), difficult to resolve using low-gradient SDS-page gels. Interestingly, the upper Foxj1 protein band diminished faster at the 2 h time point following IMD-0354 treatment (Fig. 4h, Supplementary Fig. 14). To determine if Foxj1 can be phosphorylated by IKK2, we performed co-transfection experiments using Myc-tagged Foxj1 + Flag-tagged IKK2 or IKK2-K44A kinase dead mutant^43^. When co-transfected into HEK293 cells which has low levels of endogenous IKK activity^44^, wild-type IKK2 resulted in two distinct Foxj1 protein bands, which changed to a single lower band with IKK2-K44A mutant (Fig. 4i, Supplementary Fig. 14) or IMD-0354 treatment (Fig. 4j, Supplementary Fig. 14), but not after JSH-23 treatment (Fig. 4j, Supplementary Fig. 14). Protein sequence analysis revealed a potential IKK2 phosphorylation site in Foxj1 that is highly conserved between species (Fig. 4k). Mutating the consensus serine32/35 sites in Foxj1 to alanine resulted in a lack of upper molecular weight protein band when co-transfected with IKK2 (Fig. 4l, Supplementary Fig. 14), indicating phosphorylation of Foxj1 by IKK2 activity. Test assay whether Foxj1 serine32/35 phosphorylation can stabilize the protein, we converted serine32/35 to aspartic acid, mimicking the phosphorylated state. Consistently, the aspartic acid mutations resulted in a more stable Foxj1 protein as compared to wild-type Foxj1 or the alanine mutations for serine32/35 (Fig. 4m, Supplementary Fig. 14). Co-IP experiments showed interactions between IKK2 and Foxj1, while IKK2 kinase activity and Foxj1 serine32/35 did not appear to be required for protein interactions (Fig. 4n, Supplementary Fig. 14).

### Viral-induced Foxj1 degradation and ventricular breakdown

**Fig. 5:**
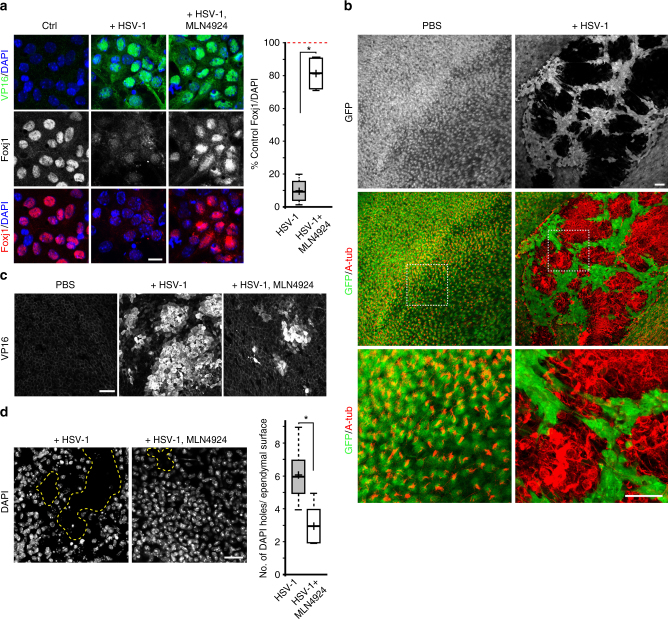
IKK-inhibiting viral degradation of Foxj1 and CSF/brain barrier disruption. **a** IHC staining of primary EC cultures without (Ctrl) or with indicated treatment conditions, labeled with Foxj1 and VP16 antibodies, and DAPI. Cultures were treated with viruses for 16 h. Quantifications showing Foxj1^+^ cells per total DAPI nuclei in treatment conditions as percentage of Ctrl condition in each experiment (read dashed line). * *P* < 0.03, Wilcoxon two-sample test, *n* = 4, *z* = 1.225. Scale bar: 15 µm. **b** IHC staining of ependymal wholemounts from P28 *FOXJ1-GFP* animals injected with PBS (Ctrl, left panels) or HSV-1 (right panels), labeled with GFP and acetylated tubulin (A-tub) antibodies. Samples were harvested 3 days after injection. Note the large GFP^+^ ependymal gaps in HSV-1 injected condition, showing ventricular wall breakdown exposing underlying brain parenchyma (A-tub^+^ cellular patches). Scale bar: 50 µm. **c** Representative IHC staining of ependymal wholemounts from P28 animals treated with PBS (Ctrl), HSV-1, or HSV-1 + MLN4924, labeled with VP16 antibody showing infected areas (VP16^+^). Samples were harvested 2 days after treatments. Scale bar: 50 µm. **d** DAPI IHC staining of ependymal wholemounts, comparing HSV-1 treated or HSV-1 + MLN4924 co-treated conditions. Image stacks represent the top 20 µm from ependymal surface. Dashed lines indicate areas of ventricular disruption where cell patches are absent from the surface. Quantifications represent average number of cellular gaps observed during each condition. * *P* < 0.0004, Wilcoxon two-sample test, *n* = 6, *z* = 1.988. Scale bar: 25 µm. Box plots show mean (+), median (−), quartiles (boxes), range (whiskers)

Having uncovered IKK2 regulation of EC Foxj1 stability, we next wanted to understand whether there are pathological conditions where IKK2 inhibition in vivo may induce hydrocephalus. There are important classes of pathogenic viruses that can suppress NF-κB activity in host cells via IKK2 inhibition^45^. HSV-1 is one such virus, which can result in devastating outcomes following CNS attack even if treated with antiviral therapy during the acute phase of infection^46^. It has been associated with hydrocephalus and ependymal ventricular wall damage^47,48^, although the underlying molecular pathogenesis for these outcomes remain largely unknown. Strikingly, within 12 h following HSV-1 infection of primary ependymal cultures, ECs uniformly lost Foxj1 protein expression (Fig. 5a). Consistent with our results above showing its effectiveness in blocking UPS-mediated Foxj1 protein turnover (Fig. 2d), MLN4924 was able to prolong EC Foxj1 protein expression and enhance cellular survival in HSV-1 infected primary ependymal cultures (Fig. 5a). Unlike HSV-1, cytomegalovirus (CMV) and Epstein-Barr virus (EBV) are two example NF-κB-activating viruses that can target the CNS^45,49,50^. We infected differentiated primary ependymal cultures with either CMV or EBV, and found no obvious changes to Foxj1 protein levels after infections (Supplementary Fig. 8a, b).

To determine whether HSV-1 can cause Foxj1 degradation in vivo, we injected HSV-1 into the lateral ventricles of P28 *FOXJ1-GFP* mice. The *FOXJ1-GFP* transgene^30^ is a useful tool for visualizing the entire ependymal lining instead of individual tamoxifen-induced R26R-tdTomato^+^ ECs. Three days after viral injection, GFP^+^ ependymal lining displayed dramatic epithelial breakdown, exposing the underlying brain parenchyma (Fig. 5b). MLN4924, similar to its effects in blunting HSV-1-induced Foxj1 degradation in primary ependymal cultures (Fig. 5a), when administered in vivo shortly after HSV-1 injection resulted in prolonged Foxj1 protein expression (Supplementary Fig. 8c), consistently reduced virally infected areas (Fig. 5c), and enhanced ventricular epithelial integrity (Fig. 5d). To examine whether induction of EC Foxj1 degradation by IKK-suppressing viruses may be generalizable, we next tested vaccinia virus which can also cause human viral encephalitis^51^. Vaccinia infection of differentiated EC cultures also resulted in Foxj1 loss, which can be blunted by MLN4924 (Supplementary Fig. 8d).

### Serum-induced Foxj1 degradation and EC transformation

**Fig. 6:**
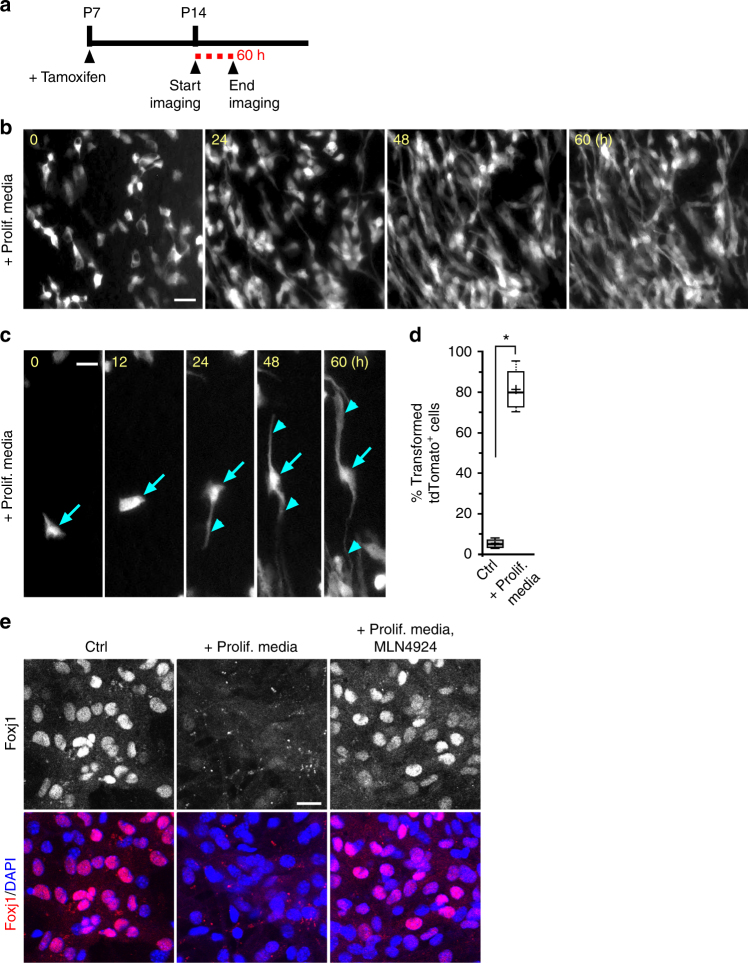
Ex vivo live imaging of mature ependymal cell transformation. **a** *foxj1*^CreERt2/+^*; R26R-tdT* mice tamoxifen induction and wholemount imaging (dashed red line) schedule. **b** Representative time-lapsed images from *foxj1*^CreERt2/+^*; R26R-tdT* ependymal wholemount sample cultured in high serum proliferation media (Prolif. media) condition. Note the cellular transformation of tdTomato^+^ ECs over time. **c** Representative close-up view of individual tdTomato^+^ EC in Prolif. media condition, showing morphological transformation over time, with radial glial-like processes (arrowheads) emanating from cell body (arrows). **d** Quantification of transformed tdTomato^+^ EC numbers for control (Ctrl) and Prolif. media conditions. *P < 0.0002, unpaired Student’s *t *test, *n* = 50, *t*_4_ = 13.732. **e** Representative IHC images of untreated (Ctrl) EC cultures; + Prolif. media; or cultured with Prolif. media then treated with MLN4924 for 12 h, labeled with Foxj1 antibody and DAPI. Scale bars: 20 µm. Box plots show mean (+), median (−), quartiles (boxes), range (whiskers)

Growth factor receptor signaling has also been shown to inhibit IKK2 activity^52,53^. During in vitro primary culture, to successfully generate high percentages of multiciliated ECs from pRGPs, we needed to change the culturing media from high (10%) to low (2%) serum. Thus we reasoned it is possible that returning mature ECs to the high serum proliferation media may induce their transformation. To test this, we injected tamoxifen in *foxj1*^CreERt2/+^*; R26R-tdT* mice at P7 and harvested ventricular wholemounts at P14 when multiciliated ECs are mature, to perform ex vivo organotypic live-imaging experiments. Unlike in control conditions where ECs retained a rather stable morphology while incubated in 2% serum culturing media, ECs incubated in 10% serum proliferation media showed a striking change in cellular morphology from epithelial to radial glial-like shape over the course of 60 h (Fig. 6a–d, Supplementary Movie 1, 2). This morphological change observed from mature tdTomato^+^ ECs was relatively uniform (Fig. 6a–d). Post imaging immunohistochemical staining showed that while tdTomato^+^ ECs in control conditions remained GFAP^−^ after 60 h of live-imaging, in proliferation media they became GFAP^+^ during their morphological transformation. Similarly, differentiated primary EC cultures treated with high serum proliferation media resulted in EC transformation and robust Foxj1 loss (Fig. 6e). MLN4924 addition to high serum proliferation media for 12 h was effective in sustaining EC Foxj1 protein expression (Fig. 6e), showing induced Foxj1 degradation via the cullin-RING ligase/UPS-dependent mechanism.

### Assessing proliferation vs. neurogenic capacity of mature ECs

**Fig. 7:**
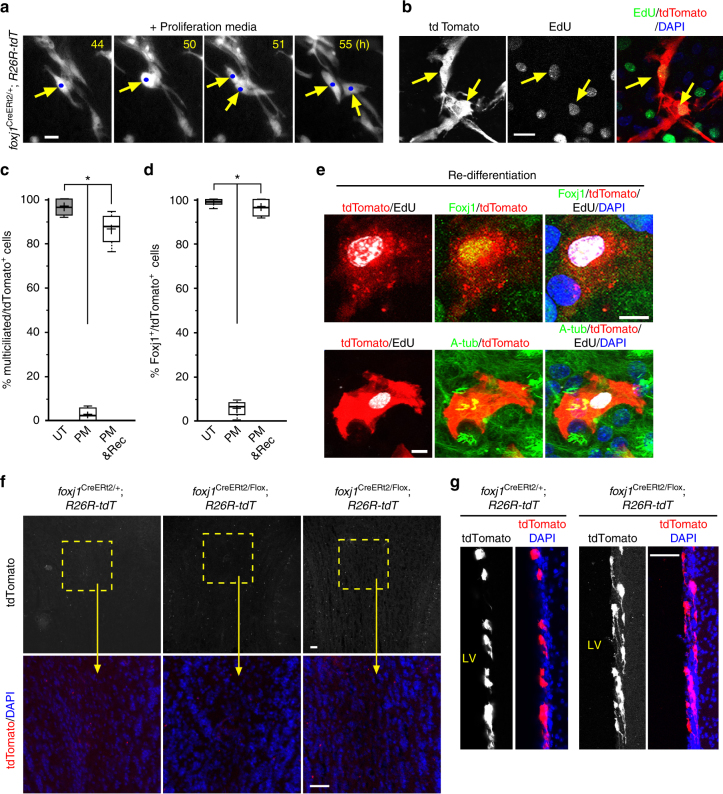
De-differentiation, cell division, and re-differentiation of mature ECs. **a** Representative time-lapsed images from P14 *foxj1*^CreERt2/+^; *R26R-tdT* ependymal wholemount sample, tamoxifen-induced at P7, cultured in proliferation media for indicated hours. Note the lineage-traced tdTomato^+^ cell (arrows) undergoing cell division (tracked by blue dots). Scale bar: 20 µm. **b** IHC staining of ependymal wholemount from P14 *foxj1*^CreERt2/+^; *R26R-tdT* animal, P7 tamoxifen-induced, cultured in proliferation media condition for 72 h, and labeled with RFP, EdU (pulsed at 36 h), and DAPI, showing EdU incorporation in lineage-traced tdTomato^+^ cells (arrows). Scale bar: 20 µm. **c** Quantification = % of lineage-traced tdTomato^+^ cells containing multicilia (marked by A-tub). *P < 0.0001, *F*_2,9_ = 15.692 (PM), one-way ANOVA, *n* *=* 4 independent cultures in all groups. UT = untreated; PM = proliferation media; Rediff = re-differentiation. Scale bar: 10 µm. **d** Quantification = % of lineage-traced tdTomato^+^ cells expressing Foxj1. *P < 0.0001, *F*_2,9_ = 12.358 (PM), one-way ANOVA, *n* *=* 4 independent cultures in all groups. **e** Representative IHC staining of mature EC cultures from *foxj1*^CreERt2/+^*;R26R-tdT* animals, tamoxifen induced in vitro, incubated with proliferation media for 4 days then re-differentiated for 10 additional days, labeled with A-tub + RFP antibodies, EdU, DAPI (upper panels) or Foxj1 + RFP antibodies, EdU, DAPI (lower panels), showing EdU^+^ re-differentiated ECs. Scale bar: 20 µm. **f**, **g** Representative IHC images of olfactory bulb (**f**) or lateral ventricle (LV) wall (**g**) sections from *foxj1*^CreERt2/+^*; R26R-tdT* or *foxj1*^CreERt2/Flox^*; R26R-tdT* animals, labeled with RFP antibody and DAPI. All animals were induced with tamoxifen in vivo at P14, and harvested: 2 weeks post induction (**f**, left and middle panels, **g**); 4 weeks post induction (**f**, right panels). Areas in dashed boxes enlarged in lower panels (**f**). Scale bars: 40 µm

The de-differentiation phenotype of mature ECs raised a question of whether they can re-enter the cell cycle. To test this, we performed live-imaging on ependymal wholemounts from P14 *foxj1*^CreERt2/+^*; R26R-tdT* mice tamoxifen-induced at P7. While we did not detect tdTomato^+^ cell divisions over 3 days of live-imaging from multiple wholemounts cultured in control media conditions (which was expected since mature ECs did not de-differentiate), when cultured in proliferation media tdTomato^+^ ECs clearly re-entered the cell cycle (Fig. 7a, Supplementary Movie 3). In support, EdU addition to the culture media to label cells entering the cell cycle consistently marked tdTomato^+^/EdU^+^ cells in proliferation media, but not control media culture conditions (Fig. 7b). Cell division of de-differentiated multiciliated ECs raised the question of what type of cells may be generated upon their re-differentiation. Since mature ECs in primary ependymal cultures also showed cell cycle re-entry and division upon proliferation media-induced de-differentiation (Supplementary Movies 4–6), and that this primary culture can robustly survive long term in vitro culture, we EdU pulsed the mature ependymal cultures concurrent with media change to either proliferation or control media incubation for 4 days. As expected following EC de-differentiation there was dramatic reduction of multiciliated/Foxj1^+^ tdTomato^+^ cells following 4 days of proliferation media incubation, unlike control media samples with abundant multiciliated ECs (Fig. 7c, d, Supplementary Fig. 9a). However, at the end of 4 days of proliferation media exposure, changing the culturing condition back to differentiation media and incubating for 10 additional days, we observed re-differentiation of lineage-traced tdTomato^+^ cells into Foxj1^+^ multiciliated ECs (Fig. 7c–e). EdU pulse-labeling of multiciliated tdTomato^+^ ECs indicated cell cycle re-entry during proliferation media-induced de-differentiation (Fig. 7e). Following re-differentiation, we did not observe tdTomato^+^/DCX^+^ neuroblasts or tdTomato^+^/GFAP^+^ astrocytes. Consistent with these findings, while Foxj1 cKO mutant ECs did not upregulate early developmental markers p73, c-Myb, Pax6, and did not show activated caspase-3 activity, they similarly became Ki67^+^ and incorporated EdU following in vivo pulse to label cells entering the cell cycle (Supplementary Fig. 9b–g). In multiple Foxj1 control littermates, we did not detect Ki67^+^ nor EdU-pulsed ECs (Supplementary Fig. 9c, f, g).

The notion that ECs can serve as a progenitor source for new neurons in the postnatal brain^15–18,33,54^ remains controversial, as other reports describe multiciliated cells as terminally differentiated^55,56^. To test whether morphologically transformed ECs without *foxj1* expression can become neurogenic, we first examined the olfactory bulbs (OBs) of *foxj1*^CreERt2/Flox^; *R26R-tdT* mutant mice injected with tamoxifen at P14, and harvested 2 and 4 weeks later. Unlike *nestin-CreER*^*tm4*^; *R26R-tdT* mice which showed robust lineage-tracing of OB interneurons post tamoxifen-induction^9,57^, we did not detect tdTomato^+^ interneurons in the OBs from multiple Foxj1 cKO mutants or littermate controls 2 or 4 weeks after tamoxifen injection (Fig. 7f, g). Cre antibody staining on P14 *foxj1*^CreERt2/+^ ventricular wholemounts showed ubiquitous Cre expression in multiciliated ECs (Supplementary Fig. 10a), giving them the capacity for tamoxifen-induced CreER recombination. While as expected two tamoxifen injections significantly increased mature ECs targeted by the *foxj1*^CreERt2/+^ driver, we did not detect tdTomato^+^ cells in the corresponding OBs 2 or 4 weeks following tamoxifen inductions (Supplementary Fig. 10b, c). Furthermore, following middle cerebral artery occlusion-induced stroke^17^ in 7-week-old mice with P14 tamoxifen induction, we did not detect lineage-traced tdTomato^+^ cells migrating from targeted ECs to the injured areas in Foxj1 cKO mutants or littermate controls (Supplementary Fig. 10d).

Studies on the cellular properties for Foxj1^+^ ECs over the last 10–15 years have relied on the human 1 kb *FOXJ1* promoter regulatory region to generate genetic and viral drivers for cellular imaging and progenitor lineage-tracing^3,4,17,33,58–61^ and/or non-specific viral driver such as CMV^16,58^. We performed the same lineage-tracing experiment via the 1 kb human promoter *FOXJ1-CreER*^*t2*^ transgenic driver^31^ crossed to *R26R-tdT* mice, injecting tamoxifen at P7 and examined 2 weeks later. We were surprised to find extensive integration of tdTomato^+^ OB interneurons (Supplementary Fig. 11a). Since we did not detect similar tdTomato labeling in the OBs of either control *foxj1*^CreERt2/+^; *R26R-tdT* nor Foxj1 cKO mice after P7 tamoxifen induction, to distinguish mislabeling of integrated OB interneurons or postnatal NSCs by the 1 kb human *FOXJ1* driver, we performed live-imaging of neuroblast migration in acute brain slice preparations from the rostral migratory stream (RMS). While we could not detect labeled cells in the RMS from *foxj1*^CreERt2/+^; *R26R-tdT* mice, we observed an abundance of migrating tdTomato^+^ neuroblasts from the *FOXJ1-CreER*^*t2*^; *R26R-tdT* RMS (Supplementary Movie 7), indicating that in addition to ECs there were mislabeled postnatal NSCs by the 1 kb human *FOXJ1* promoter. In support, the widely used *FOXJ1-GFP* reporter mouse line^30^ (utilizing the same 1 kb human regulatory element), in addition to labeling ECs also expressed GFP in Foxj1-negative cells in the postnatal lateral ventricular niche (Supplementary Fig. 11b). Unlike at P7 and older ages where CreER and Foxj1 proteins are co-localized in *foxj1*^CreERt2/+^ mice, at P0 we detected numerous CreER^+^ but Foxj1-negative lateral ventricular pRGPs (Supplementary Fig. 11c). Following P0 tamoxifen injection in *foxj1*^CreERt2/+^; *R26R-tdT* animals, at P21 we observed tdTomato^+^ OB interneurons, as well as tdTomato^+^ lateral ventricular progenitors without Foxj1 protein expression nor multicilia (Supplementary Fig. 11d). With P0 tamoxifen injection, P10 live-imaging of RMS showed migrating tdTomato^+^ neuroblasts (Supplementary Fig. 11d), consistent with CreER-targeting of some neural progenitors transiently upregulating *foxj1* transcription at this stage (thus expressing CreER but without stabilizing Foxj1 protein required for EC differentiation). We did not observe lineage-traced neurogenic capacity at any time point following P7 or P14 tamoxifen injection in *foxj1*^CreERt2/+^; *R26R-tdT* mice.

Recent results suggested that CD133^+^ ECs can give rise to neuroblasts postnatally^18,54^; thus there may exist a neurogenic multiciliated ependymal population that does not express Foxj1. We examined this possibility by searching for Foxj1-negative multiciliated ECs in *nestin-Cre; foxj1*^KO/Flox^ mutant mice^4^, which restrict *foxj1* deletion to the CNS. We reasoned that multiciliated ECs remaining in these mutant mice would be the best candidates. In ventricular wholemount preparations from P10 *nestin-Cre; foxj1*^KO/Flox^ mutant mice, while as expected most of the ventricular surface lacked multicilia, we were able to find occasional multiciliated cells (Supplementary Fig. 12a). IHC staining revealed that these multiciliated cells uniformly expressed Foxj1, as well as CD133 (Supplementary Fig. 12b, c). These results showed that the remaining multiciliated ECs in *nestin-Cre; foxj1*^KO/Flox^ mutant mice were Foxj1^+^ ECs which escaped Cre-mediated recombination. We did not detect the existence of Foxj1-negative multiciliated ECs.

## Discussion

The development and functions of multiciliated brain ECs, generally believed to be a terminally differentiated cell type, are poorly understood. To identify pathways that modulate ependymal differentiation/maturation, we uncovered an inherent cellular plasticity in mature ECs. We found that the Foxj1 transcription factor is required to maintain the multiciliated phenotype of ECs, but this important protein has a short half-life, constantly targeted for UPS-mediated degradation. To identify key molecular drivers leading to ependymal Foxj1 degradation, transcriptome and biochemical analyses revealed a non-canonical IKK2-dependent activity regulating Foxj1 protein stability. Armed with these molecular insights, we demonstrated that this previously undescribed signaling pathway is directly targeted by known pathological inhibitors of IKK2 to induce EC Foxj1 degradation and brain ventricular surface breakdown. Since the ependyma provides important barrier functions^62^ and directly contacts the CSF, which is frequently used to diagnose CNS diseases, we believe these results will have important implications for hydrocephalus pathophysiology and neuro-immune interactions.

ECs form a highly specialized epithelium lining the brain ventricles, with a distinct cellular feature of multiple motile cilia emanating from their apical surfaces. The ependymal developmental program involves sequential specialization of this multiciliated phenotype, including rapid duplication of basal bodies^63^, followed by upregulation of the Foxj1 transcription factor to induce motile multiciliary development, as well as morphological change from a glial to epithelial cell type^3,4^. Supposed to last the lifetime of the animal, there was little experimental evidence to suggest that mature ECs retained the ability to divide and self-renew, which will require the disassembly of multiple basal bodies and their associated motile cilia. While these cellular specializations may, in principle, indicate that mature ECs represent a stably differentiated cell population, our findings here showed that the continuous production of a single transcription factor, Foxj1, is necessary to actively prevent phenotypic transformation of mature ECs. We demonstrated that Foxj1 is constantly turned over via UPS-mediated degradation, and its removal from mature ECs resulted in de-differentiation. These results bring to mind the “Red Queen Hypothesis”, where differentiated ECs are constantly running to stand still by making and stabilizing Foxj1, whose degradation then allows ECs to re-enter cell cycle and regenerate. It will be of interest to unravel the signaling networks controlling Foxj1 protein stability in mature ECs.

To determine which biological processes may accelerate ependymal Foxj1 degradation, we performed transcriptome and biochemical analyses, and uncovered, quite unexpectedly, that a non-canonical IKK2 activity was required to stabilize Foxj1 protein. While IKK2 phosphorylation of NF-κB inhibitor IκB is well known, other IKK phosphorylation sites have been identified in proteins such as IRS-1 and Foxo3a^64,65^, with amino acid sequence similarities to serines 32/35 motifs in Foxj1. This IKK2 target site on Foxj1 is highly conserved evolutionarily from frogs, rodents, to human. Given the importance of IKK pathway function in diverse pathological processes, it was reasonable to assume that there may be pathological/disease states inducing IKK2 suppression in ECs. Growth factor exposure and pathogenic viruses have been previously shown to inhibit IKK2 activity^52,53^, and were thus good candidates for investigation. HSV-1 is an IKK inhibiting virus that can result in devastating outcomes following CNS attack, particularly in infants^46^. It has been associated with ventricular wall damage and hydrocephalus, although the underlying molecular pathogenesis for these outcomes remain largely unknown. Ventricular bleeding is another major cause of hydrocephalus^14^. Whereas obstruction of CSF flow from blood clots is a known initiator, serum factors aberrantly introduced into CSF may also disrupt ventricular integrity. Remarkably, we found that both viral infections and serum treatment can robustly induce ependymal Foxj1 degradation and EC de-differentiation. Considering that inheritable human mutations resulting in hydrocephalus are relatively rare, environmental exposures that tilt EC Foxj1 protein towards sustained degradation provide additional avenues to explore for understanding and treating acquired cases of hydrocephalus.

Consistent with our initial observation that MLN4924 was able to stabilize Foxj1 protein by inhibiting UPS-targeted degradation, in vitro/in vivo applications of MLN4924 showed attenuation of HSV-1-induced EC Foxj1 degradation and ventricular wall disruption. MLN4924 is currently in clinical trials as an anti-cancer treatment^23^ due to its effectiveness in blocking cell cycle progression^22^. While MLN4924 has a beneficial effect on preserving ependymal Foxj1 and ventricular integrity, it will likely blunt the important adaptive immune responses where proliferation of microglia, astrocytes, and peripheral immune cells are needed to restore tissue homeostasis. SMER3, while not a specific chemical inhibitor of mammalian Fbxw cullin-RING ligases, has been shown to have selective activity against related homolog SCFMET30 in yeast^26^. Developing similar class of compounds to target specific mammalian Fbwx ligase family members may promote EC Foxj1 protein stability while sparing cell cycle inhibition.

Once we observed that Foxj1 loss could induce de-differentiation and cell division in mature ECs, we became hopeful that this step may be the long-sought molecular prelude to new neuron production, and a link to unite divergent views in the adult neurogenesis field. To test this, we first examined the OBs of *foxj1*^CreERt2/Flox^; *R26R-tdT* mutant mice following postnatal tamoxifen injection. While we did not detect tdTomato^+^ OB interneurons in control *foxj1*^CreERt2/+^*; R26R-tdT* mice after tamoxifen induction, we reasoned that any tdTomato^+^ OB interneurons found in the conditional mutant mice would indicate ependymal neurogenic capacity following *foxj1* removal. At either 2 or 4-week time points following tamoxifen injection, we did not detect tdTomato^+^ OB interneurons in *foxj1*^CreERt2/Flox^*; R26R-tdT* mutant mice. We observed similar negative results following cortical stroke. Robust numbers of tdTomato^+^ ECs from the lateral ventricles of these experimental animals ruled out lack of tamoxifen-induced recombination as a potential confound.

Previous studies tackling neurogenic properties of multiciliated ECs have depended on non-specific CMV viral drivers^16,58^, or the human 1 kb *FOXJ1* promotor regulatory element to generate reporters and Cre drivers^3,17,33,58–61^. While Ad-CMV-Cre also targets postnatal neural stem cells effectively^66,67^, in principle lineage-tracing strategies utilizing the *FOXJ1* promoter should be EC-specific. Repeating the same tamoxifen-inducible experiment in *FOXJ1-CreER*^*t2*^*; R26R-tdT* mice, we found robust tdTomato labeling of OB interneurons. Live-imaging of migrating tdTomato^+^ neuroblasts in *FOXJ1-CreER*^*t2*^*; R26R-tdT* mice, as well as *FOXJ1-GFP* expression in Foxj1-negative cells, revealed non-specific labeling of neurogenic population by the human 1 kb *FOXJ1* promotor. While it remains possible that de-differentiated mature ECs can become neurogenic in other contexts, our results here indicate a cellular plasticity program for cell cycle re-entry and EC renewal.

## Methods

### Knockout and transgenic lines

All mouse experiments were performed according to an approved protocol by the Institutional Animal Care and Use Committee at Duke University. The following lines were used: *foxj1*^Flox/+^ ^4^; *FOXJ1-GFP*^30^; *FOXJ1-CreER*^*t2*^ ^31^; *nestin-Cre*^68^. To generate the *foxj1*^CreERt2/+^ driver, CreER^t2^ sequence was inserted in-frame into ATG start codon of the *foxj1* gene. The knock-in targeting construct was made by standard BAC recombineering techniques, electroporated into mouse embryonic stem cells by standard methods. PCR genotyping primers: P1 (TGG CTT GCA GGT ACA GGA GG), P2 (CCT CAT TGT GGC CAA CAA ACC C); 94 °C 30 s, 60 °C 1 min, 72 °C 1 min, 32 cycles. Tamoxifen (10 mg/ml, freshly dissolved in corn oil) was injected intraperitoneally, 0.15 mg per gram of body weight to induce recombination.

### DNA constructs

The Foxj1-Myc plasmid was described previously and contains a C-terminal Myc-tag downstream of the Foxj1 ORF^4^. The shRNA sequence for Fbxw5 (CCATACAACTGGAGTTACA) was cloned into the plvthm plasmid. The Fbxw5 construct contains an N-terminal 3× -Myc tag and is cloned into pcDNA3 plasmid. The pRK5-HA-Ubiquitin-WT containing an N-terminal HA tag was acquired from addgene.com. pCR-IKK2(Wt)-Flag and pCR-IKK2(K44A) mutant was acquired from addgene.com. Lentiviral constructs containing validated mouse shRNAs against IKK2 or a scrambled control were purchased from Geneocopia. Site-directed mutagenesis kit (New England Biolabs) was used to convert Foxj1 serines 32 and 35 to aspartic acid or alanine residues. Inserts containing the mutations of interest were sub-cloned into PWPXLD plasmid and sequenced to ensure no off-target mutations.

### Primary ependymal cultures and chemical screen

ECs were grown in 24-well dishes from isolated radial glial progenitors as previously described^4^. For chemical screen, we used pathway selective custom library purchased from Selleck Chemicals. For immunofluorescence staining, cell cultures were grown on 12 mm glass coverslips, washed once in PBS then fixed in either 2% or 4% paraformaldehyde solution. Fixed cells were permeabilized in PBS containing 0.1% TritonX-100 (PBST) and blocked using 10% donkey serum in PBST. Primary antibody solutions were incubated with cells overnight at 4 °C and conjugated secondary antibodies were incubated for 3 h at 4 °C. DAPI was incubated for 20 min at room temperature and coverslips were placed onto slides with aqua-poly/mount for confocal imaging. During differentiation MLN4924 was included during the switch to differentiation media at 0.25 µM and incubated for 2 days, then sequentially halved with differentiation media for 3 additional days. SMER3 was incubated in mature ependymal cultures at a final concentration of 5 µM for 12 h. MG132 was incubated with ependymal cultures at a final concentration of 25 µM in ependymal differentiation media. Cycloheximide was used at a final concentration of 20 µg/ml in ependymal differentiation media. Ependymal cultures were incubated with JSH-23 and SC75741 at a final concentration of 25 and 5 µM, respectively. For EC re-differentiation after de-differentiation, EC cultures made from *foxj1*^CreER/+^; *R26R-tdT* animals were: (1) fully differentiated as described above, and CreER-dependent tdTomato expression was achieved by treating cultures with 4-OH-tamoxifen (1 µg/ml, Sigma) for 24 h, followed by 7 days of culturing in differentiation media. (2) The EC cultures were then treated with proliferation media for 4 days, EdU (1 µg/ml, Sigma) was added for the last 2 days. (3) Culture was switched back to differentiation media for further 10 days prior to analyses.

### Quantitative PCR

Total RNA was extracted from EC cultures using RNA easy mini Kit (Qiagen) and cDNA was prepared using Superscript Vilo cDNA synthesis kit (Invitrogen). Quantitative PCR analysis was performed using SYBR Green as previously described^57^ using primer sets Foxj1 F: 5′-tcactctgtcggccatctac-3′ and R: 5′-ttcttgaaggccccactgag-3′; GAPDH F: 5′-catggccttccgtgttcct-3′ and R: 5′- tgatgtcatcatacttggcaggtt-3′.

### Immunohistochemistry and imaging

IHC of brain coronal sections were prepared as previously described^9^. Brain ventricular lateral wall wholemounts were isolated as previously described^4^ and fixed directly in paraformaldehyde solution (4 or 2%) followed by permeabilization in 0.1% PBST solution. Donkey serum at 10% in PBST was used to block tissue sections and wholemounts prior to immunolabeling. All images were acquired on Leica TCS SP5 confocal microscope, with control and experimental samples imaged under identical settings. Primary antibodies solutions were incubated overnight with tissue and fluorescently conjugated secondary were incubated for 3 h and 4 °C. For treatment of wholemounts with MLN4924, cycloheximide, and IMD0354 drugs, tissue was incubated in 0.5 µM, 25 µg/ml, and 0.5 µM concentration respectively for the indicated lengths of time. The following antibodies and dilutions were used: Foxj1 (rabbit, 1:1000, Sigma); Acetylated-tubulin (mouse, 1:1000, Sigma); VP16 (mouse, 1:1000, Santa Cruz); anti-CMV (mouse, 1:1000, Millipore); JF186/EBV (mouse, 1:500, gift of Micah Luftig); anti-vaccinia virus (mouse, 1:1000, Abcam); Fbxw5 (goat, 1:500, Santa Cruz); RFP (rabbit, 1:1000, Rockland); Acetyl-tubulin (mouse, 1:1000, Sigma); GLAST (mouse, 1:1000, Novus); DCX (guinea pig, 1:200, Millipore); CD31 (mouse, 1:1000, Invitrogen); HA (mouse, 1:1000, Genescript); Myc (mouse, 1:1000, thermo-scientific); Cre (mouse, 1:250, Millipore); Mcidas (rabbit, 1:250, Biorbyt); p73 (mouse, 1:250, Novus Biologicals); PAX6 (mouse, 1:250, abcam); Ki67 (rabbit, 1:1000, abcam); IKK2 (mouse, 1:1000 blot, Novus Biologicals); cyclin D1 (mouse, 1:2000, BD Biosciences); BIII-tubulin (mouse, 1:1000, R&D Systems); c-Myb (mouse, 1:250, Santa Cruz); Activated-Caspase 3 (rabbit, 1:250, Cell Signaling). Live-imaging was performed as previously described^57^, using upright Leica SP8 confocal microscope. For organotypic cultures, lateral ventricle ependymal wholemounts were isolated as previously described^4^, placed on collagen-coated membrane inserts (Millipore), incubated in control media (DMEM/F12, 2% FBS, 1% glutamine, and 1% Pen/Strep) or proliferation media (DMEM/F12, 10% FBS, EGF (20 ng/ml), bFGF (20 ng/ml), 1% glutamine, and 1% Pen/Strep), and live-imaged on Zeiss Cell Observer® System equipped with incubation system set at 37 °C and 5% CO_2_. For 3D ependymal morphological quantification: confocal z-stack images of tdTomato^+^ ECs were first reconstructed in Imaris 3D view, and number of major basal extensions quantified via Imaris 3D filament tracer software module. Ependymal soma position was used as start point, and protrusions <5 µm were excluded from analyses to focus on significant cellular extensions.

### Biochemistry and immunoprecipitation

Cell cultures or tissue samples were lysed and prepared for immunoblotting as described previously^4,69^. Lysates were resolved on Tris-Glycine SDS-PAGE gels (Bio-Rad) and transferred onto PVDF membranes (Bio-Rad). Antibodies were diluted in PBS containing 0.1% Triton-X100 with 2% BSA, followed by overnight incubation at 4 °C. Detection was accomplished through secondary antibodies conjugated to horseradish peroxidase (Cell Signaling) and treated with enhanced chemiluminescence. To separate the Foxj1 doublet lysates were allowed to migrate through premade 4–20% gradient Tris-Glycine gels (Bio-Rad) for 1.5 h at 170 volts. Immunoprecipitations were performed on ependymal cultures lysed in buffer containing 0.02 M Hepes buffer, 0.15 M NaCl, 1% Triton-X100, sodium orthovanadate (Sigma), protease inhibitor cocktail (Roche), 10 mM NaF. Supernatants were collected from lysates spun at 15 K rpm for 20 min, and primary antibodies incubated either 4 h or overnight at 4 °C. Secondary antibodies conjugated to agarose beads were incubated for 2 h at 4 °C. Beads were washed five times in lysis buffer, harvested in 5× PAGE, processed for SDS-PAGE analysis and western blotting. HEK293 cells were grown on 24-well culture dishes. Twenty-four hours after plating HEK293 cells were transfected with equal amounts of plasmids for Foxj1-Myc-WT, IKK2-WT, or IKK2 mutant. Twenty-four hours later transfection media was replaced with low serum media, then harvested 16 h later for SDS-PAGE and western blotting. To separate the Foxj1 doublet lysates were allowed to migrate through premade 4–20% gradient Tris-Glycine gels (Bio-Rad) for 1.5 h at 170 volts. The following antibodies and dilutions were used for the study: Foxj1 (rabbit, 1:2000, Sigma); Flag (mouse, 1:2000, Sigma); HA (mouse, 1:2000, Genescript); Myc (mouse, 1:2000, Thermo-Scientific); ubiquitin (mouse, 1:2000, IMGENEX); Fbxw5 (goat, 1:2000, Santa Cruz); actin (rabbit, 1:5000, Sigma).

### Viral preparation

Lentiviruses were produced in 293T17 cells through triple-transfection of transfer plasmids (pLVTHM or pWPXLD), psPAX2, and pMD2.G. Lentivirus containing media was collected, filtered through 0.45 micrometer membranes. To concentrate lentiviruses media was spun for 2 h at 25 K rpm using a swinging bucket rotor and a Beckman Coulter ultracentrifuge. For HSV-1, vero cells were grown to confluence on 10 cm tissue culture dishes in DMEM supplemented with 10% fetal bovine serum at 37 °C in 5% CO_2._ Cells were infected with HSV-1 KOS strain^70^. After 48 h media containing HSV-1 virus was collected, cleared through centrifugation, and filtered through 0.45 micrometer pore membranes and stored at −80 °C. Viral titers were determined through plaque assay of Vero cells. HSV-1-KOS was maintained at a stock of 10^7^ plaque-forming units/ml.

### In vitro and in vivo viral infections

ECs were cultured in 24-well plates and cells were infected with an MOI of 2 for HSV-1-KOS or vaccinia virus. After 4 h media was replaced and cells assayed at the time points indicated. For HSV-1 and MLN4924 co-treatment, ECs were treated with HSV-1-KOs for 4 h then treated with MLN4924. MLN4924 was added 24 h after initial treatment with vaccinia virus. CMV virus and EBV virus were incubated with ependymal cultures for 4 h and media was replaced and ECs cultured for an additional 4 days. For in vivo infections mice were anesthetized by gas phase isoflurane mixed with oxygen. HSV-1-KOS virus in 0.12 µl (2×10^4^ pfu) was injected intraventricularly through stereotaxic parameters in 3–4-week-old mice similarly to previously described method^4^. Coordinates for intraventricular injections (zeroed from bergma): *X* = 0.9 µm, *Y* = 2.5 µm, *Z* = 2.2 µm. For MLN4924 drug treatment mice were first given MLN4924 intraventricularly at a final concentration of 0.5 µM. This was followed shortly by an injection of HSV1 within the same injection site. Mice were assayed at the time points indicated. To assay barrier breakdown DAPI^+^ nuclei were used to measure gaps on the ependymal surface.

### In vivo injections and injury

For in vivo EdU labeling, 1 mg of EdU (in 100 µl of saline) was administered through intraperitoneal injection. Seven-week-old anesthetized animals were induced for focal ischemic brain injury through middle cerebral artery occlusion as described^17^.

### Transcriptome analyses

Affymetrix Gene Chip microarray data underwent strict quality control processing using the “simpleaffy” package in the Bioconductor 2 suite from the R statistical programming environment. Log-scale Robust Multiarray Analysis from the “affy” package was used to normalize the data and eliminate systematic differences across the arrays. Differential expression of genes across the conditions was identified from a moderated test statistic employed by the “limma” package. The False Discovery Rate (FDR) method was used to correct for multiple hypothesis testing. Gene set enrichment analysis was performed to identify differentially regulated pathways and gene ontology terms for each of the comparisons performed. Heatmap of the differentially expressed genes (FDR ≤ 0.05) for each 〈Condition〉 vs. 〈Condition〉 comparison. Gene expression has been *z*-score normalized and the samples and genes are clustered by correlation distance with complete linkage. The heatmap shows all genes that were identified as significant in at least one of the comparisons. Heatmaps only include genes that had an FDR ≤ 0.05 and a log2FC >1 or <−1. Hierarchical clustering plot of all of the samples based on expression of all genes on the arrays was shown. The samples are clustered using a correlation distance with complete linkage. Samples are colored by their condition.

### Statistical analysis

No statistical methods were used to pre-determine samples sizes, but they are similar to those reported elsewhere^4,9,57^. For representative images and western blots, the results were shown to be reproducible by at least three separate experiments. Blocking of experimental design was assigned by animal genotype and was not randomized. Depending on sample size, unpaired Student’s *t* test (≥10) or Wilcoxon two-sample test (≥5) were used for statistical comparisons between two data sets. Throughout, box-and-whisker plots were generated via http://boxplot.tyerslab.com/ to depict mean (+), median (line), low and high quartiles (boxes), and range (whiskers).

### Data availability

Microarray data are available from the GEO database, accession number GSE111182. All other remaining data are available within the article and Supplementary Files, or available from the authors upon request.

## Electronic supplementary material

Supplementary Information

Description of Additional Supplementary Files

Supplementary Movie 1

Supplementary Movie 2

Supplementary Movie 3

Supplementary Movie 4

Supplementary Movie 5

Supplementary Movie 6

Supplementary Movie 7

## References

[1] Del Bigio MR (2010). Ependymal cells: biology and pathology. Acta Neuropathol..

[2] Spassky N (2005). Adult ependymal cells are postmitotic and are derived from radial glial cells during embryogenesis. J. Neurosci..

[3] Jacquet BV (2009). FoxJ1-dependent gene expression is required for differentiation of radial glia into ependymal cells and a subset of astrocytes in the postnatal brain. Development.

[4] Paez-Gonzalez P (2011). Ank3-dependent SVZ niche assembly is required for the continued production of new neurons. Neuron.

[5] Tan FE (2013). Myb promotes centriole amplification and later steps of the multiciliogenesis program. Development.

[6] Gonzalez-Cano L (2016). p73 is required for ependymal cell maturation and neurogenic SVZ cytoarchitecture. Dev. Neurobiol..

[7] Kyrousi C, Lalioti ME, Skavatsou E, Lygerou Z, Taraviras S (2016). Mcidas and GemC1/Lynkeas specify embryonic radial glial cells. Neurogenesis.

[8] Tramontin AD, Garcia-Verdugo JM, Lim DA, Alvarez-Buylla A (2003). Postnatal development of radial glia and the ventricular zone (VZ): a continuum of the neural stem cell compartment. Cereb. Cortex.

[9] Kuo CT (2006). Postnatal deletion of Numb/Numblike reveals repair and remodeling capacity in the subventricular neurogenic niche. Cell.

[10] Guirao B (2010). Coupling between hydrodynamic forces and planar cell polarity orients mammalian motile cilia. Nat. Cell. Biol..

[11] Mirzadeh Z, Han YG, Soriano-Navarro M, Garcia-Verdugo JM, Alvarez-Buylla A (2010). Cilia organize ependymal planar polarity. J. Neurosci..

[12] Tissir F (2010). Lack of cadherins Celsr2 and Celsr3 impairs ependymal ciliogenesis, leading to fatal hydrocephalus. Nat. Neurosci..

[13] Ohata S (2014). Loss of Dishevelleds disrupts planar polarity in ependymal motile cilia and results in hydrocephalus. Neuron.

[14] Zhang J, Williams MA, Rigamonti D (2006). Genetics of human hydrocephalus. J. Neurol..

[15] Chiasson BJ, Tropepe V, Morshead CM, van der Kooy D (1999). Adult mammalian forebrain ependymal and subependymal cells demonstrate proliferative potential, but only subependymal cells have neural stem cell characteristics. J. Neurosci..

[16] Johansson CB (1999). Identification of a neural stem cell in the adult mammalian central nervous system. Cell.

[17] Carlen M (2009). Forebrain ependymal cells are Notch-dependent and generate neuroblasts and astrocytes after stroke. Nat. Neurosci..

[18] Luo Y (2015). Single-cell transcriptome analyses reveal signals to activate dormant neural stem cells. Cell.

[19] Lam EW, Brosens JJ, Gomes AR, Koo CY (2013). Forkhead box proteins: tuning forks for transcriptional harmony. Nat. Rev. Cancer.

[20] Alten L (2012). Differential regulation of node formation, nodal ciliogenesis and cilia positioning by Noto and Foxj1. Development.

[21] Brody SL, Yan XH, Wuerffel MK, Song SK, Shapiro SD (2000). Ciliogenesis and left-right axis defects in forkhead factor HFH-4-null mice. Am. J. Respir. Cell Mol. Biol..

[22] Soucy TA (2009). An inhibitor of NEDD8-activating enzyme as a new approach to treat cancer. Nature.

[23] Swords RT (2015). Pevonedistat (MLN4924), a First-in-Class NEDD8-activating enzyme inhibitor, in patients with acute myeloid leukaemia and myelodysplastic syndromes: a phase 1 study. Br. J. Haematol..

[24] Nurtjahja-Tjendraputra E, Fu D, Phang JM, Richardson DR (2007). Iron chelation regulates cyclin D1 expression via the proteasome: a link to iron deficiency-mediated growth suppression. Blood.

[25] Nagle AA (2012). Induction of tumor cell death through targeting tubulin and evoking dysregulation of cell cycle regulatory proteins by multifunctional cinnamaldehydes. PLoS ONE.

[26] Aghajan M (2010). Chemical genetics screen for enhancers of rapamycin identifies a specific inhibitor of an SCF family E3 ubiquitin ligase. Nat. Biotechnol..

[27] Gao J (2014). Nuclear retention of Fbw7 by specific inhibitors of nuclear export leads to Notch1 degradation in pancreatic cancer. Oncotarget.

[28] Kanei-Ishii C, Nomura T, Egoh A, Ishii S (2012). Fbxw5 suppresses nuclear c-Myb activity via DDB1-Cul4-Rbx1 ligase-mediated sumoylation. Biochem. Biophys. Res. Commun..

[29] Kim TY (2015). Substrate trapping proteomics reveals targets of the betaTrCP2/FBXW11 ubiquitin ligase. Mol. Cell. Biol..

[30] Ostrowski LE, Hutchins JR, Zakel K, O’Neal WK (2003). Targeting expression of a transgene to the airway surface epithelium using a ciliated cell-specific promoter. Mol. Ther..

[31] Rawlins EL, Ostrowski LE, Randell SH, Hogan BL (2007). Lung development and repair: contribution of the ciliated lineage. Proc. Natl. Acad. Sci. USA.

[32] Zhang Y (2007). A transgenic FOXJ1-Cre system for gene inactivation in ciliated epithelial cells. Am. J. Respir. Cell Mol. Biol..

[33] Meletis K (2008). Spinal cord injury reveals multilineage differentiation of ependymal cells. PLoS Biol..

[34] Doetsch F, Caille I, Lim DA, Garcia-Verdugo JM, Alvarez-Buylla A (1999). Subventricular zone astrocytes are neural stem cells in the adult mammalian brain. Cell.

[35] Petroski MD, Deshaies RJ (2005). Function and regulation of cullin-RING ubiquitin ligases. Nat. Rev. Mol. Cell Biol..

[36] Milhollen MA (2010). MLN4924, a NEDD8-activating enzyme inhibitor, is active in diffuse large B-cell lymphoma models: rationale for treatment of NF-{kappa}B-dependent lymphoma. Blood.

[37] Perkins ND (2007). Integrating cell-signalling pathways with NF-kappaB and IKK function. Nat. Rev. Mol. Cell Biol..

[38] Tanaka A (2005). A novel NF-kappaB inhibitor, IMD-0354, suppresses neoplastic proliferation of human mast cells with constitutively activated c-kit receptors. Blood.

[39] Hacker H, Karin M (2006). Regulation and function of IKK and IKK-related kinases. Sci. Stke..

[40] Greten FR (2007). NF-kappaB is a negative regulator of IL-1beta secretion as revealed by genetic and pharmacological inhibition of IKKbeta. Cell.

[41] Pasparakis M (2002). TNF-mediated inflammatory skin disease in mice with epidermis-specific deletion of IKK2. Nature.

[42] Chariot A (2009). The NF-kappaB-independent functions of IKK subunits in immunity and cancer. Trends Cell Biol..

[43] Nakano H (1998). Differential regulation of IkappaB kinase alpha and beta by two upstream kinases, NF-kappaB-inducing kinase and mitogen-activated protein kinase/ERK kinase kinase-1. Proc. Natl. Acad. Sci. USA.

[44] Xiao G (2001). Retroviral oncoprotein Tax induces processing of NF-kappaB2/p100 in T cells: evidence for the involvement of IKKalpha. EMBO J..

[45] Hiscott J, Kwon H, Genin P (2001). Hostile takeovers: viral appropriation of the NF-kappaB pathway. J. Clin. Invest..

[46] Corey L, Wald A (2009). Maternal and neonatal herpes simplex virus infections. N. Engl. J. Med..

[47] Hayashi K, Iwasaki Y, Yanagi K (1986). Herpes simplex virus type 1-induced hydrocephalus in mice. J. Virol..

[48] Conrady CD (2013). Microglia and a functional type I IFN pathway are required to counter HSV-1-driven brain lateral ventricle enlargement and encephalitis. J. Immunol..

[49] DeMeritt IB, Milford LE, Yurochko AD (2004). Activation of the NF-kappaB pathway in human cytomegalovirus-infected cells is necessary for efficient transactivation of the major immediate-early promoter. J. Virol..

[50] Fujimoto H (2003). Epstein-Barr virus infections of the central nervous system. Intern. Med..

[51] Garcel A, Fauquette W, Dehouck MP, Crance JM, Favier AL (2012). Vaccinia virus-induced smallpox postvaccinal encephalitis in case of blood−brain barrier damage. Vaccine.

[52] Drafahl KA, McAndrew CW, Meyer AN, Haas M, Donoghue DJ (2010). The receptor tyrosine kinase FGFR4 negatively regulates NF-kappaB signaling. PLoS ONE.

[53] Wei W (2012). The breast cancer susceptibility gene product fibroblast growth factor receptor 2 serves as a scaffold for regulation of NF-kappaB signaling. Mol. Cell. Biol..

[54] Coskun V (2008). CD133+ neural stem cells in the ependyma of mammalian postnatal forebrain. Proc. Natl. Acad. Sci. USA.

[55] Marshall WF, Kintner C (2008). Cilia orientation and the fluid mechanics of development. Curr. Opin. Cell Biol..

[56] Brooks ER, Wallingford JB (2014). Multiciliated cells. Curr. Biol..

[57] Benner EJ (2013). Protective astrogenesis from the SVZ niche after injury is controlled by Notch modulator Thbs4. Nature.

[58] Nomura T, Goritz C, Catchpole T, Henkemeyer M, Frisen J (2010). EphB signaling controls lineage plasticity of adult neural stem cell niche cells. Cell Stem Cell.

[59] Sabelstrom H (2013). Resident neural stem cells restrict tissue damage and neuronal loss after spinal cord injury in mice. Science.

[60] Jacquet BV (2011). Specification of a Foxj1-dependent lineage in the forebrain is required for embryonic-to-postnatal transition of neurogenesis in the olfactory bulb. J. Neurosci..

[61] Devaraju K, Barnabe-Heider F, Kokaia Z, Lindvall O (2013). FoxJ1-expressing cells contribute to neurogenesis in forebrain of adult rats: evidence from in vivo electroporation combined with piggyBac transposon. Exp. Cell Res..

[62] Shook BA (2014). Ventriculomegaly associated with ependymal gliosis and declines in barrier integrity in the aging human and mouse brain. Aging Cell.

[63] Stubbs JL, Vladar EK, Axelrod JD, Kintner C (2012). Multicilin promotes centriole assembly and ciliogenesis during multiciliate cell differentiation. Nat. Cell Biol..

[64] Hu MC (2004). IkappaB kinase promotes tumorigenesis through inhibition of forkhead FOXO3a. Cell.

[65] Gao Z (2002). Serine phosphorylation of insulin receptor substrate 1 by inhibitor kappa B kinase complex. J. Biol. Chem..

[66] Harms KM, Li L, Cunningham LA (2010). Murine neural stem/progenitor cells protect neurons against ischemia by HIF-1alpha-regulated VEGF signaling. PLoS ONE.

[67] Ventura RE, Goldman JE (2007). Dorsal radial glia generate olfactory bulb interneurons in the postnatal murine brain. J. Neurosci..

[68] Tronche F (1999). Disruption of the glucocorticoid receptor gene in the nervous system results in reduced anxiety. Nat. Genet..

[69] Paez-Gonzalez P, Asrican B, Rodriguez E, Kuo CT (2014). Identification of distinct ChAT(+) neurons and activity-dependent control of postnatal SVZ neurogenesis. Nat. Neurosci..

[70] Jurak I, Silverstein LB, Sharma M, Coen DM (2012). Herpes simplex virus is equipped with RNA- and protein-based mechanisms to repress expression of ATRX, an effector of intrinsic immunity. J. Virol..

