# Supplementary material for: Uncovering inherent cellular plasticity of multiciliated ependyma leading to ventricular wall transformation and hydrocephalus
Source: Nat Commun. 2018 Apr 25;9:1655. doi: 10.1038/s41467-018-03812-w (PMC5916891; doi:10.1038/s41467-018-03812-w)
Supplement: Supplementary file 1 — Supplementary Information [file 41467_2018_3812_MOESM1_ESM.pdf]

Supplementary Figures and Legends

Uncovering inherent cellular plasticity of multiciliated ependyma leading to ventricular wall transformation and hydrocephalus

Abdi et al.

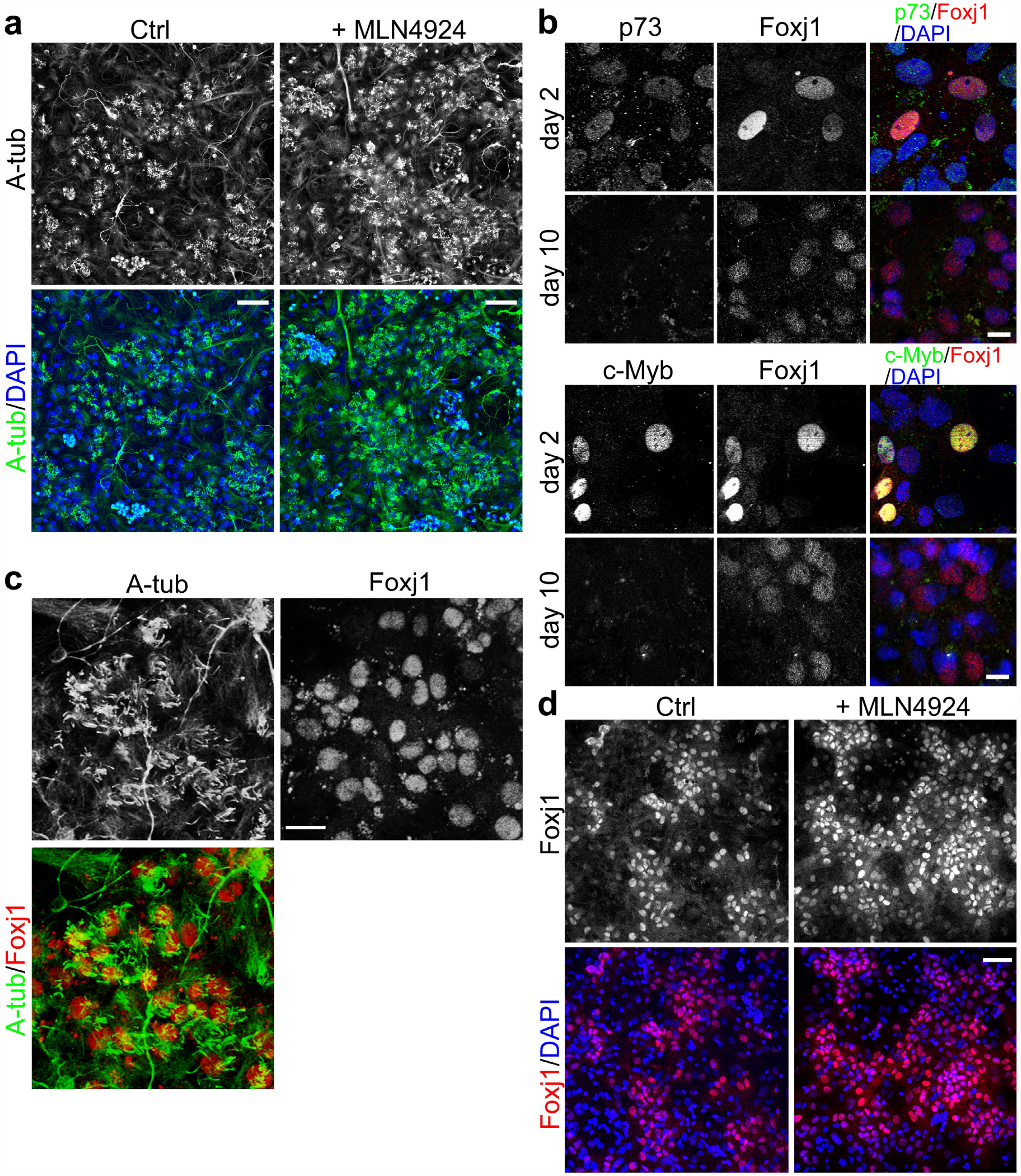

Supplementary Figure 1 (**a**,**d**) Representative confocal images showing large field views of primary EC cultures without (Ctrl) or with MLN4924 added during cellular differentiation from progenitors. IHC staining for acetylated tubulin (A-tub) + DAPI (**a**), or Foxj1 + DAPI (**d**). Scale bars: 50 μm. (**b**) IHC staining of primary EC cultures, showing representative p73 and c-Myb co-expression with Foxj1 during early differentiation (day 2) but not in mature ECs (day 10). Scale bar: 10 μm. (**c**) Representative IHC staining of mature primary EC cultures showing uniform Foxj1 expression in multiciliated cells (visualized by Acetylated-tubulin, A-tub). Scale bar: 20 μm.

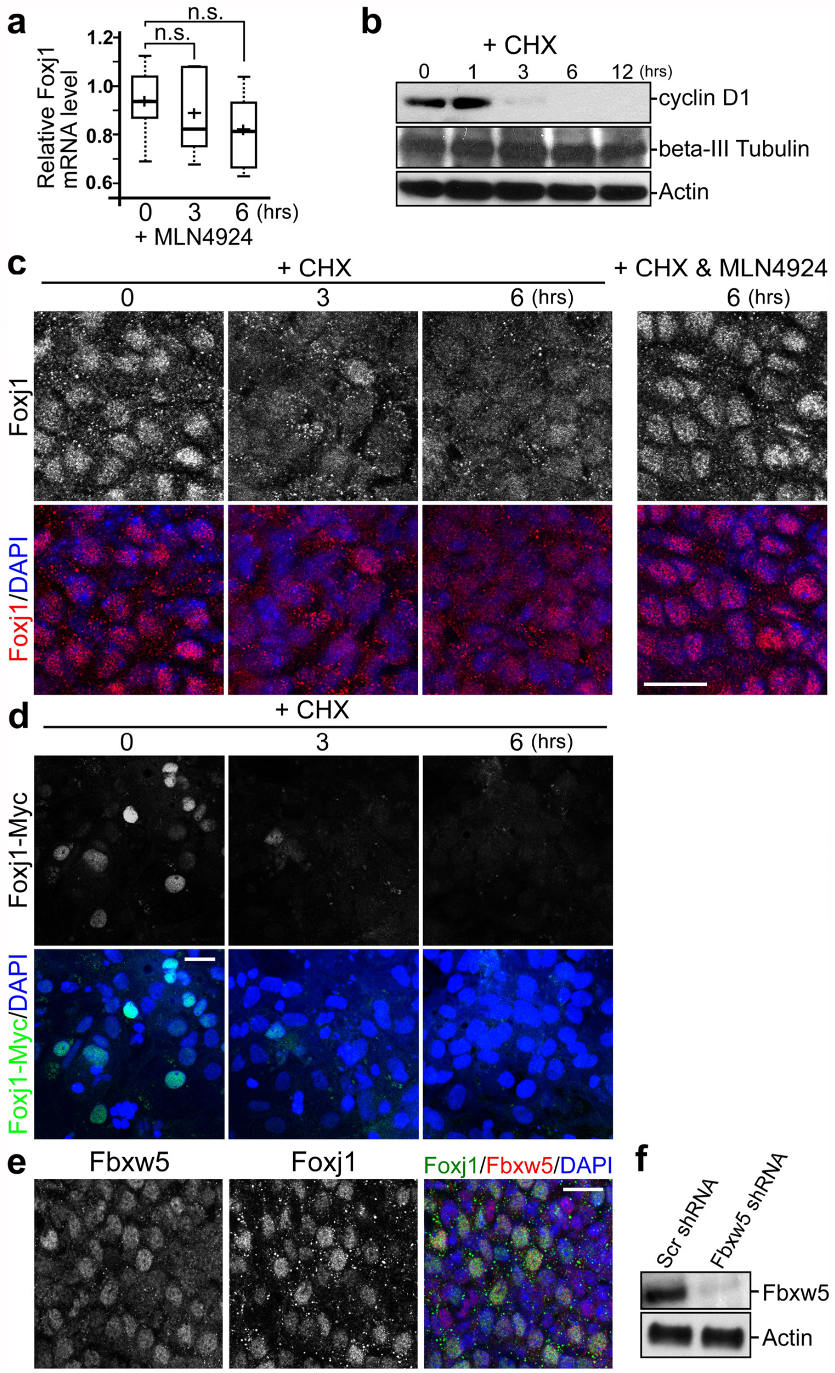

Supplementary Figure 2 (**a**) Quantitative PCR analyses of Foxj1 mRNA levels in MLN4924-treated primary EC cultures for indicated lengths of time. Wilcoxon 2-sample test, n = 5 cultures. Box plots show mean (+), median (–), quartiles (boxes), range (whiskers). (**b**) Western blots of primary EC culture lysates treated with CHX for the indicated lengths of time, and probed with cyclin D1 and beta-III Tubulin antibodies. Anti-Actin was used as loading control. (**c**) Confocal images from P28 ependymal wholemounts treated with cycloheximide (CHX) for indicated lengths of time, IHC staining for Foxj1 and DAPI. Right panels: sustained ependymal Foxj1 expression when MLN4924 is added with cycloheximide. (**d**) IHC staining of primary EC cultures expressing Foxj1-Myc cDNA for 5 days followed by CHX treatment for indicated lengths of time, with anti-Myc antibody and DAPI. (**e**) IHC staining of P28 brain ependymal wholemount with antibodies to Foxj1, Fbxw5, and DAPI. Note consistent overlap of Fbxw5 expression with Foxj1. (**f**) Western blots of protein lysates from primary EC cultures treated with lentiviral scrambled control (Scr) or Fbxw5 shRNA, and probed with anti-Foxj1 antibody. Anti-Actin was used as loading control. Scale bars: 20 μm.

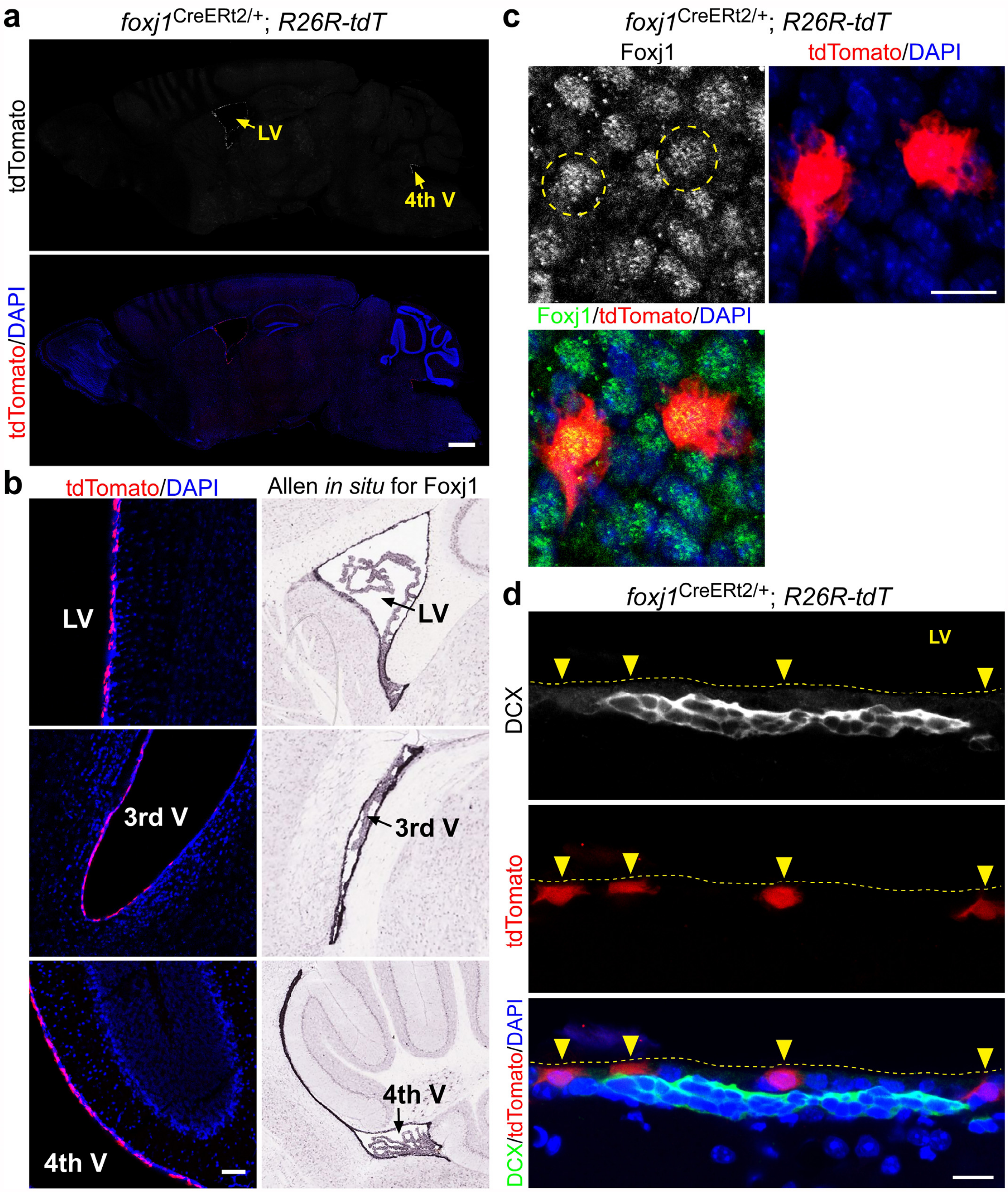

Supplementary Figure 3 (**a**) Representative sagittal brain section from P30 *foxj1*$^{CreERt2/+}$; *R26R-tdT* animal tamoxifen induced at P14, labeled with RFP antibody and DAPI, showing tdTomato expression around lateral ventricle (LV) and 4th ventricle (4th Vent). Scale bar: 1 mm. (**b**) IHC staining of coronal brain sections from P30 *foxj1*$^{CreERt2/+}$; *R26R-tdT* animals, tamoxifen induced at P14, labeled with RFP antibody and DAPI, showing ependymal-specific tdTomato expression in the LV, 3rd ventricle (3rd V), and 4th V. Representative Foxj1 in situ expression data from Allen Brain Atlas is shown in corresponding right panels. Scale bars: 60 μm. (**c**) Representative confocal image of ependymal wholemount from P28 *foxj1*$^{CreERt2/+}$*; R26R-tdT* animal, IHC staining for Foxj1, tdTomato, and DAPI, showing Foxj1 expression in tdTomato^{+} ECs (dashed circles). (**d**) IHC staining of lateral ventricular wall section from P28 *foxj1*$^{CreERt2/+}$*; R26R-tdT* animal stained for DCX, tdTomato, and DAPI, showing lack of co-localization between tdTomato (arrowheads) and DCX signals. Scale bars: 15 μm.

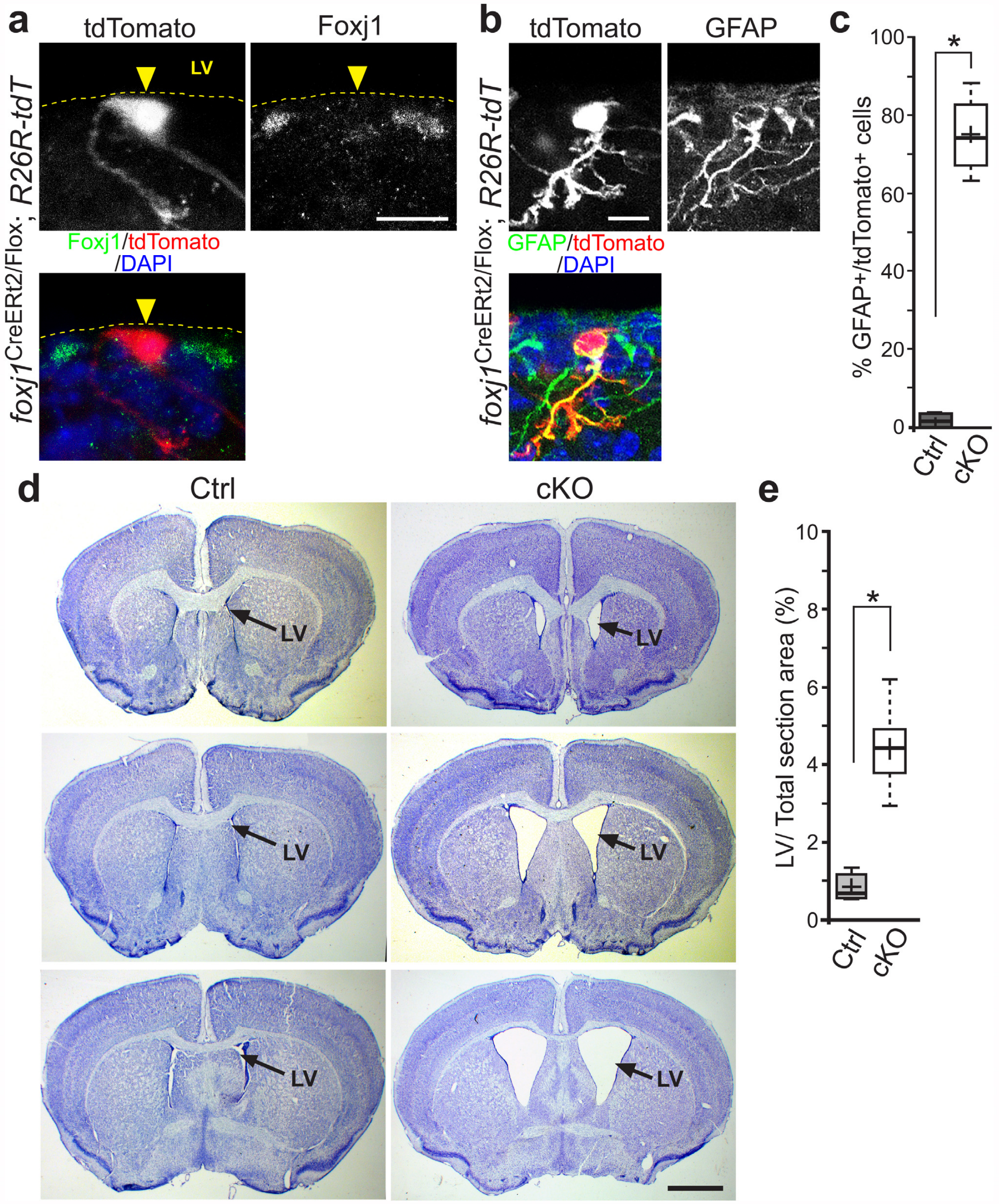

Supplementary Figure 4 (**a**) IHC staining of lateral ventricular wall section from P28 *foxj1*$^{CreERt2/Flox}$*; R26R-tdT* mutant animal, stained for tdTomato, Foxj1, and DAPI, showing lack of Foxj1 expression in tdTomato^{+} ependymal cell (arrowhead). P14 tamoxifen induction in all experiments shown. LV = lateral ventricle. Scale bars: 15 µm. (**b**) Representative IHC staining of brain section from P28 *foxj1*$^{CreERt2/Flox}$; *R26R-tdT* animal, tamoxifen induced at P14, labeled with RFP + GFAP antibodies, and DAPI showing GFAP expression in tdTomato^{+} Foxj1 mutant EC. Scale bar: 20 µm. (**c**) Quantification of tdTomato^{+} Foxj1 mutant ECs expressing GFAP. * $P < 0.03$, Wilcoxon 2-sample test, $n = 4$ mice in each group, $z = 1.320$. (**d**) Nissl staining of brain sections from P28 *foxj1*$^{Flox/+}$*; R26R-tdT* (Ctrl) and *foxj1*$^{CreERt2/Flox}$*; R26R-tdT* (cKO) mice tamoxifen-induced at P14. Note the increased lateral ventricle (LV) size in cKO animals. (**e**) Quantitative analyses of LV size: % of LV area over total brain section area (to control for variations in section sizes during processing), using coronal sections covering anterior commissure. * $P < 0.008$, Wilcoxon 2-sample test, $n = 5$ mice in each group, $z = 1.526$. Scale bar: 1 mm. Box plots show mean (+), median (–), quartiles (boxes), range (whiskers).

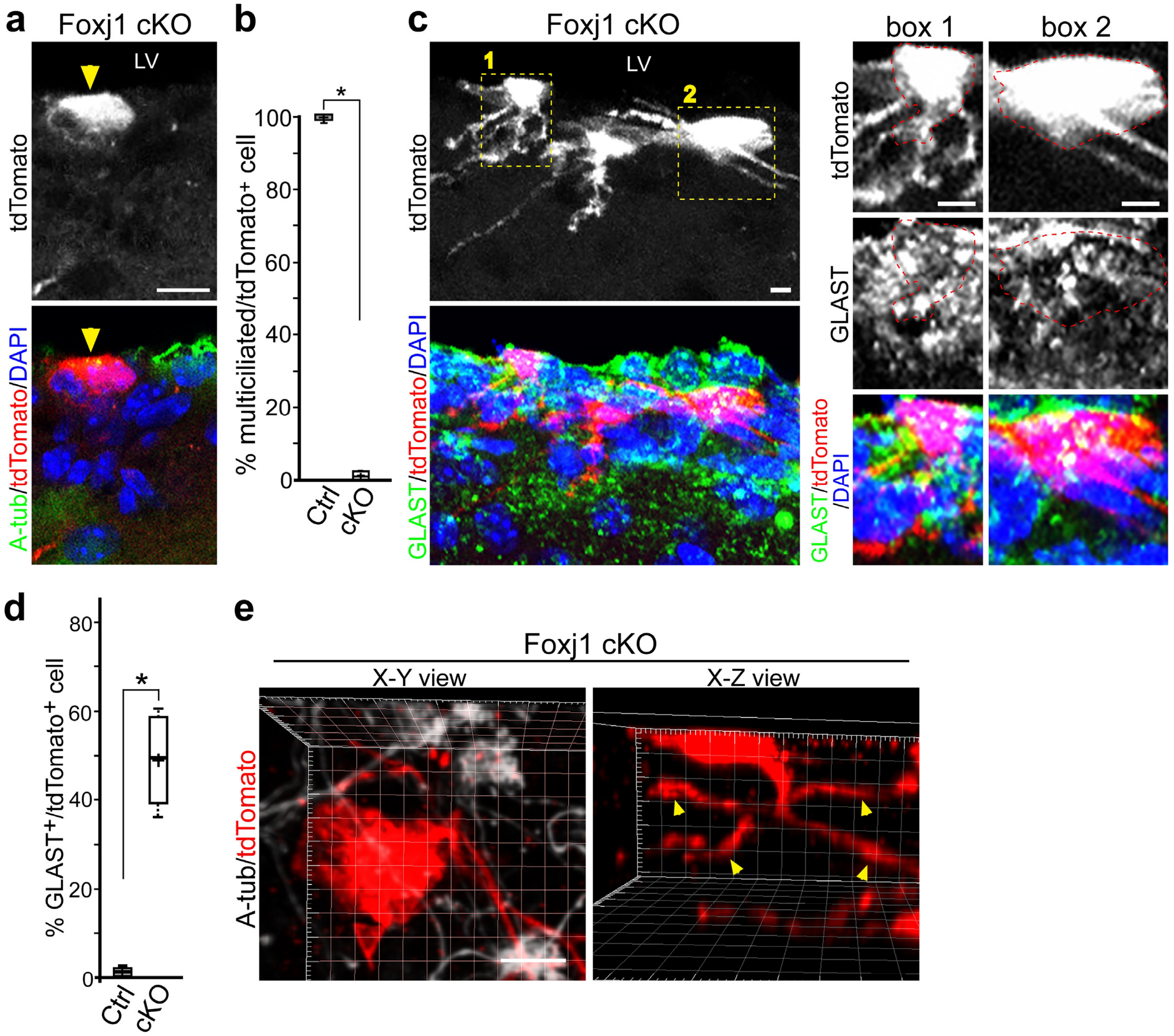

Supplementary Figure 5 (**a-e**) *foxj1*$^{CreERt2/Flox}$; *R26R-tdT* (cKO) animals tamoxifen induced at P30 and analyzed 2 weeks later. (**a**) Representative IHC staining of lateral ventricle (LV) brain sections labeled with A-tub + RFP antibodies, and DAPI. Note the lack of multicilia in lineage-traced Foxj1 cKO tdTomato^{+} ECs. Scale bar: 10 µm. (**b**) Quantification = % of tdTomato^{+} ECs showing A-tub^{+} multicilia, comparing cKO and littermate controls (Ctrl). * $P < 0.03$, Wilcoxon 2-sample test, n = 4 mice, z = 1.408. (**c**) IHC staining of LV brain sections labeled with GLAST + RFP antibodies, and DAPI. Right panels represent enlarged views of boxes 1 and 2 in left panels (dashed yellow lines). GLAST upregulation in Foxj1 mutant ECs seen as intracellular aggregates (dashed red lines). Scale bars: 5 µm. (**d**) Quantification = % of tdTomato^{+} ECs co-labeling with GLAST. * $P < 0.03$, Wilcoxon 2-sample test, n = 4 mice, z = 1.226. (**e**) Imaris 3D rendering of tdTomato^{+} lineage-traced Foxj1 cKO ECs, labeled with A-tub (white) and RFP (red) antibodies. X-Y (left panel) and X-Z (right panel) views illustrate morphological changes of Foxj1 cKO ECs extending basal processes (arrowheads). Scale bar: 10 µm. Box plots show mean (+), median (–), quartiles (boxes), range (whiskers).

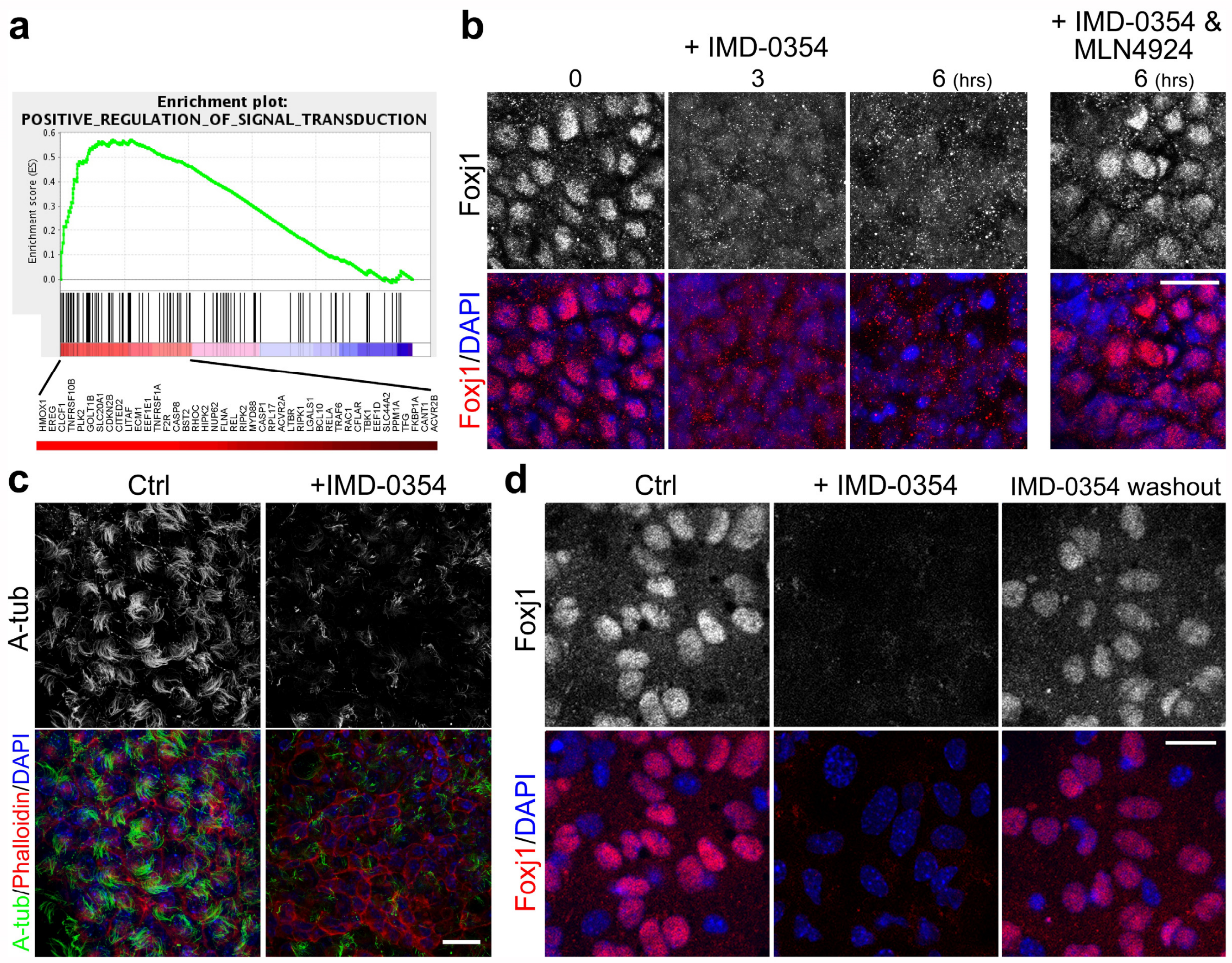

Supplementary Figure 6 (**a**) Transcriptome analyses of MLN4924's effects on primary EC cultures: 'positive regulation of signal transduction' pathway gene enrichment plot, via gene set enrichment analysis (GSEA) using gene ontology database. Genes were ranked based on significance and direction of expression change (*t*-statistic). There are significant gene overlaps between this pathway and the IKK/NK-κB signaling pathway. (**b**) IHC staining of P28 ependymal wholemounts, treated with IMD-0354 for indicated lengths of time, with anti-Foxj1 antibody and DAPI, showing Foxj1 protein loss following incubation, which can be blunted by MLN4924 (far right panels). (**c**) IHC staining of primary EC cultures untreated or treated with IMD-0354 for 48 hours, labeled with Foxj1 + Acetylated-tubulin (A-tub) antibodies, and DAPI. (**d**) IHC staining of primary EC cultures with anti-Foxj1 antibody and DAPI: untreated (Ctrl), treated with IMD-0354 for three days (middle panels), or treated with IMD-0354 for three days followed by washout/recovery for an additional three days (right panels). Scale bars: 20 μm.

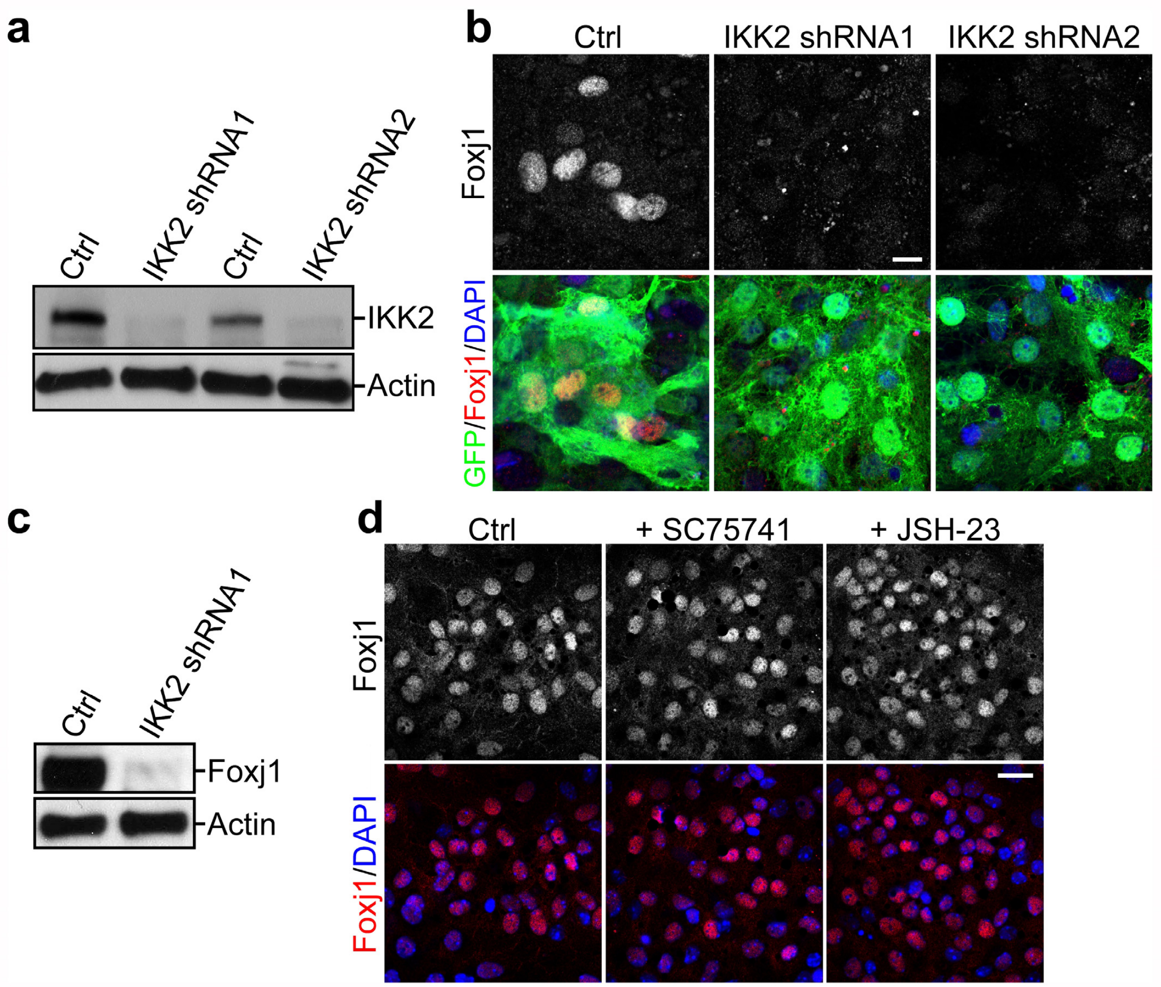

Supplementary Figure 7 (**a**) Western blot analysis of primary EC culture lysates treated with either control (Ctrl) or IKK2 shRNAs, and probed with antibodies against IKK2 and Actin, showing IKK2 protein knockdown. (**b**) Representative IHC staining of primary EC cultures treated with either scrambled control (Ctrl) or IKK2 shRNAs, labeled with GFP (showing infected cells) + Foxj1 antibodies, and DAPI. Note the downregulated Foxj1 protein expression following IKK2 knockdown. Scale bar: 10 μm. (**c**) Western blot analysis of primary EC culture lysates treated with Ctrl or IKK2 shRNA construct, probed with Foxj1 and Actin antibodies. (**d**) IHC staining of primary EC cultures with anti-Foxj1 antibody and DAPI: untreated (Ctrl), treated with NF-κB inhibitors SC75741 or JSH-23 for 24 hours. Note that SC75741 and JSH-23 did not induce Foxj1 protein loss. Scale bars: 20 μm.

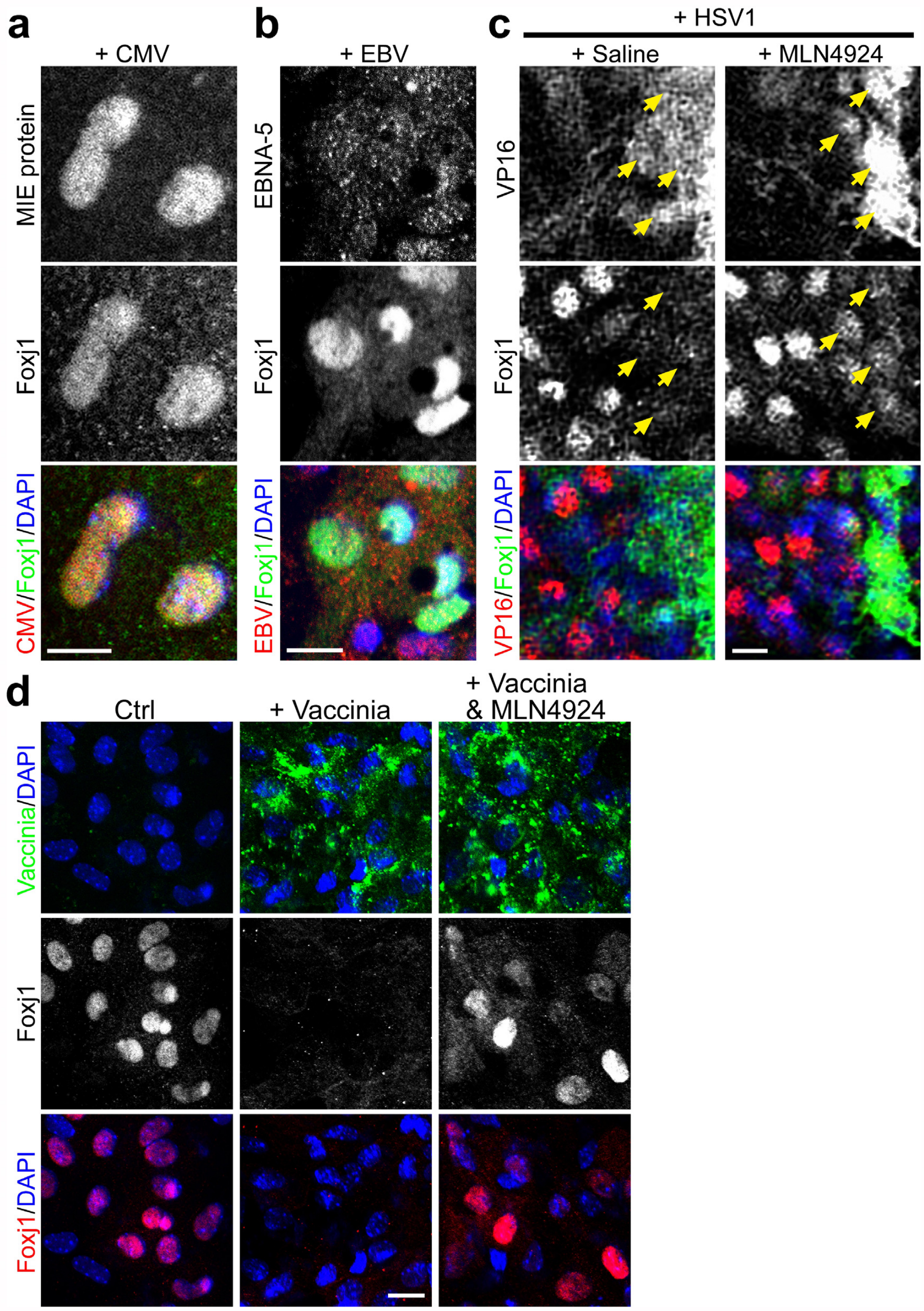

Supplementary Figure 8 (**a**,**b**) Representative IHC staining images of primary EC cultures infected with CMV (a) or EBV (**b**), with antibodies against CMV + Foxj1 (**b**), EBNA-5 + Foxj1 (**b**), and DAPI. (**c**) Confocal IHC staining images of ependymal wholemounts harvested from animals following HSV-1 injection into the lateral ventricles with either saline (left panels) or MLN4924 (right panels), using antibodies for VP16, Foxj1, and DAPI. P28 animals were injected with HSV-1 and harvested 24 hours later. Arrows point to VP16^{+} infected ependymal cells. (**d**) IHC staining of primary EC cultures in PBS (Ctrl), vaccinia virus, or vaccinia virus + MLN4924 treated conditions, labeled with Foxj1 and vaccinia virus antibodies, and DAPI. Cultures were treated with viruses for 24 hours. Note that vaccinia virus-induced downregulation of Foxj1 can be blunted by MLN4924. Scale bars: 10 μm.

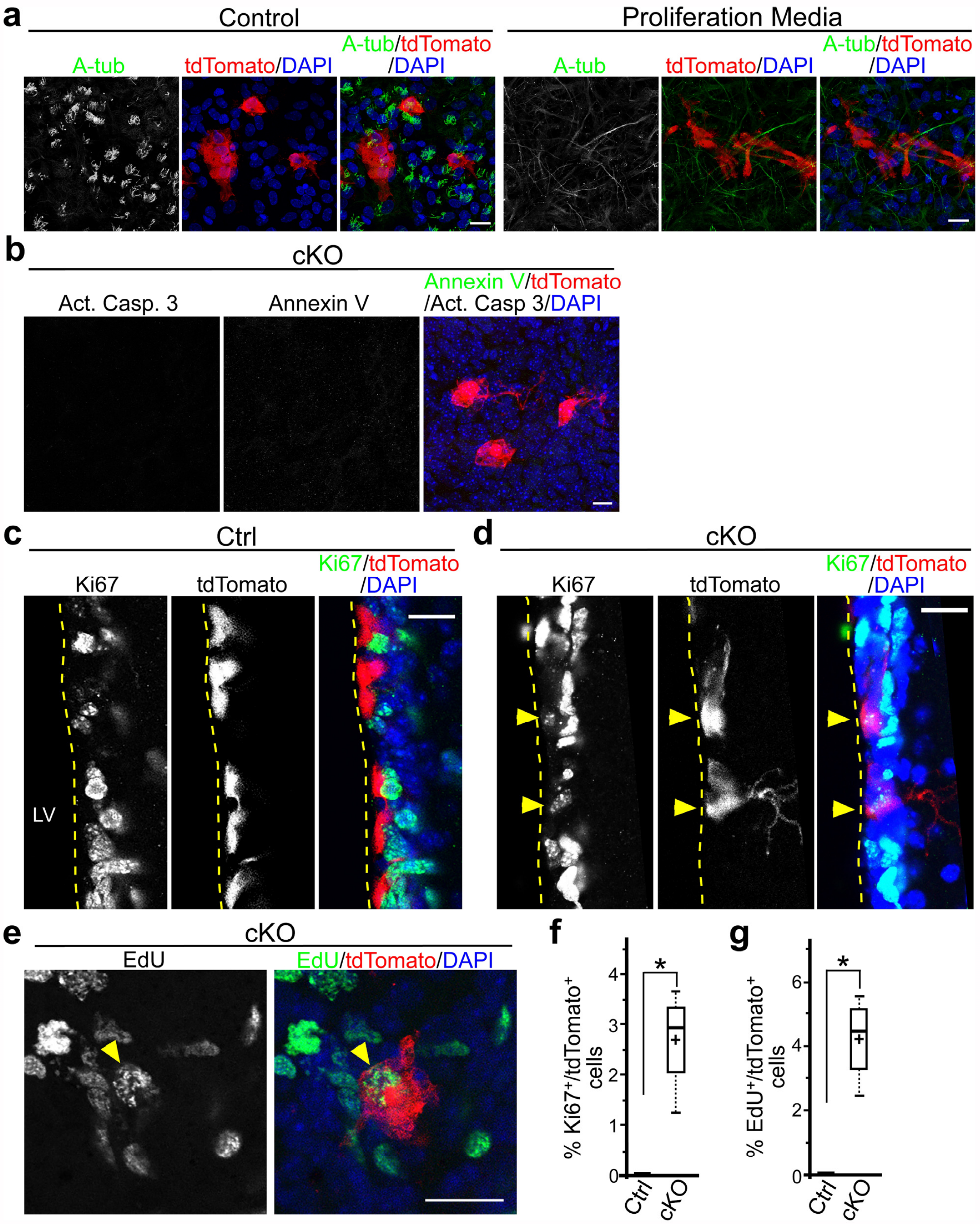

Supplementary Figure 9 (**a**) Representative IHC staining of tamoxifen-induced mature EC cultures from *foxj1*$^{CreERt2/+}$*; R26R-tdT* animal, untreated (control) or treated with Proliferation Media for 4 days, labeled with A-tub + RFP antibodies, and DAPI. Scale bar: 20 μm. (**b**) Representative IHC staining of ependymal wholemounts from P28 *foxj1*$^{CreERt2/Flox}$; *R26R-tdT* (cKO) animals tamoxifen injected at P14, labeled with FITC-Annexin V, anti-activated Caspase 3 antibody, and DAPI, showing lack of apoptosis in tdTomato^{+} lineage-traced ECs. Scale bar: 20 μm. (**c**,**d**) Representative IHC staining of coronal brain sections from P28 littermate control (Ctrl) (**c**) and Foxj1 cKO (**d**) animals, tamoxifen-induced at P14, labeled with Ki67 + RFP antibodies, and DAPI, showing Ki67^{+} lineage-traced cKO tdTomato^{+} ECs (arrowheads). Scale bar: 20 μm. (**e**) IHC staining of P28 Foxj1 cKO ependymal wholemount, tamoxifen-induced at P14, labeled with RFP antibody, EdU, and DAPI, showing representative EdU^{+} lineage-traced Foxj1 cKO EC (arrowheads). Scale bar: 20 μm. (**f**) Quantification = % of lineage-traced tdTomato^{+} cells co-labeling with Ki67. * $P < 0.03$, Wilcoxon 2-sample test, $n = 4$ mice, $z = 1.095$. (**g**) Quantification = % of lineage-traced tdTomato^{+} cells showing EdU incorporation. * $P < 0.03$, Wilcoxon 2-sample test, $n = 4$ mice, $z = 1.156$. Box plots show mean (+), median (–), quartiles (boxes), range (whiskers).

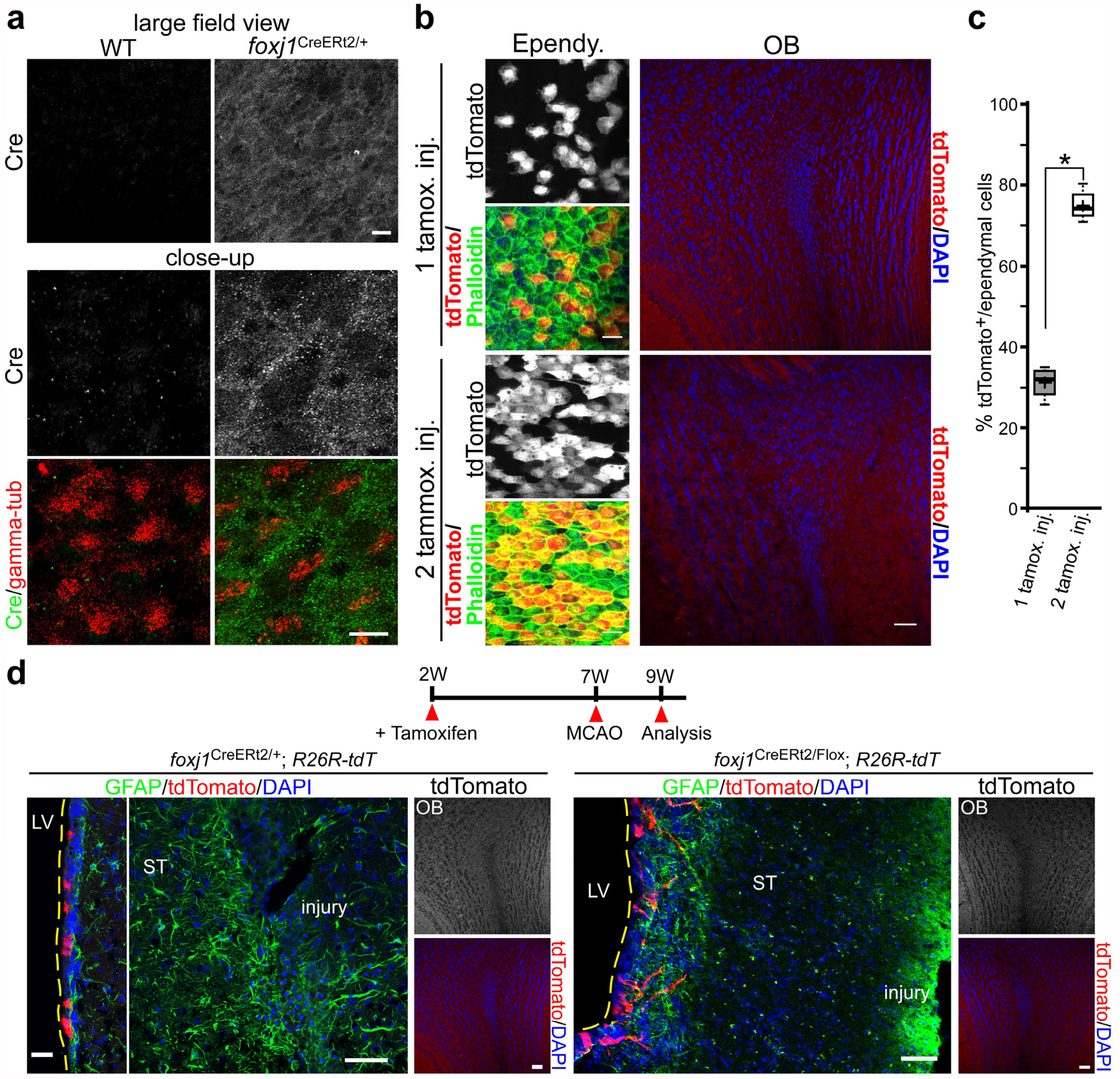

Supplementary Figure 10 (**a**) IHC staining of ependymal wholemounts from P28 wild type (WT) and *foxj1*$^{CreERt2/+}$ animals, labeled with Cre and gamma-tubulin (gamma-tub) antibodies, demonstrating abundant CreER expression in multiciliated ECs from *foxj1*$^{CreERt2/+}$ driver. Images were acquired at identical confocal settings. Scale bars: 20 μm, (upper panels), 10 μm (lower panels). (**b**) Representative IHC staining of ependymal wholemounts and corresponding olfactory bulb (OB) sections from P28 *foxj1*$^{CreERt2/+}$; *R26R-tdT* animals tamoxifen injected at P14 (1 tamox. inj.), or P14 & P16 (2 tamox. inj.), labeled with RFP antibody, phalloidin, and DAPI. Note the increased ependymal labeling with 2 tamoxifen injections (lower panels), and lack of lineage-traced OB tdTomato^{+} cells under either conditions. Scale bar: 20 μm (ependy), 60 μm (OB). (**c**) Quantification = % of ECs (epithelial outlined by phalloidin) becoming tdTomato^{+} after 1 or 2 tamoxifen injections. * $P < 0.03$, Wilcoxon 2-sample test, $n = 4$ mice, $z = 1.4077$. Box plots show mean (+), median (–), quartiles (boxes), range (whiskers). (**d**) IHC staining of brain sections from *foxj1*$^{CreERt2/+}$; *R26R-tdT* (left panels) and *foxj1*$^{CreERt2/Flox}$; *R26R-tdT* (right panels) animals following MCAO-induced injury, with experimental time course indicated by illustration. Coronal brain sections with lateral ventricle (LV) niche and injured striatum (ST) were labeled with GFAP + RFP antibodies, and DAPI (left panels), showing lack of lineage-traced tdTomato^{+} cells at the injured areas. Scale bar: 60 μm. Right panels representing corresponding OB sections, stained with RFP antibody and DAPI, showing a lack of lineage-traced tdTomato^{+} cells in both genotypes after injury. Scale bars: 60 μm.

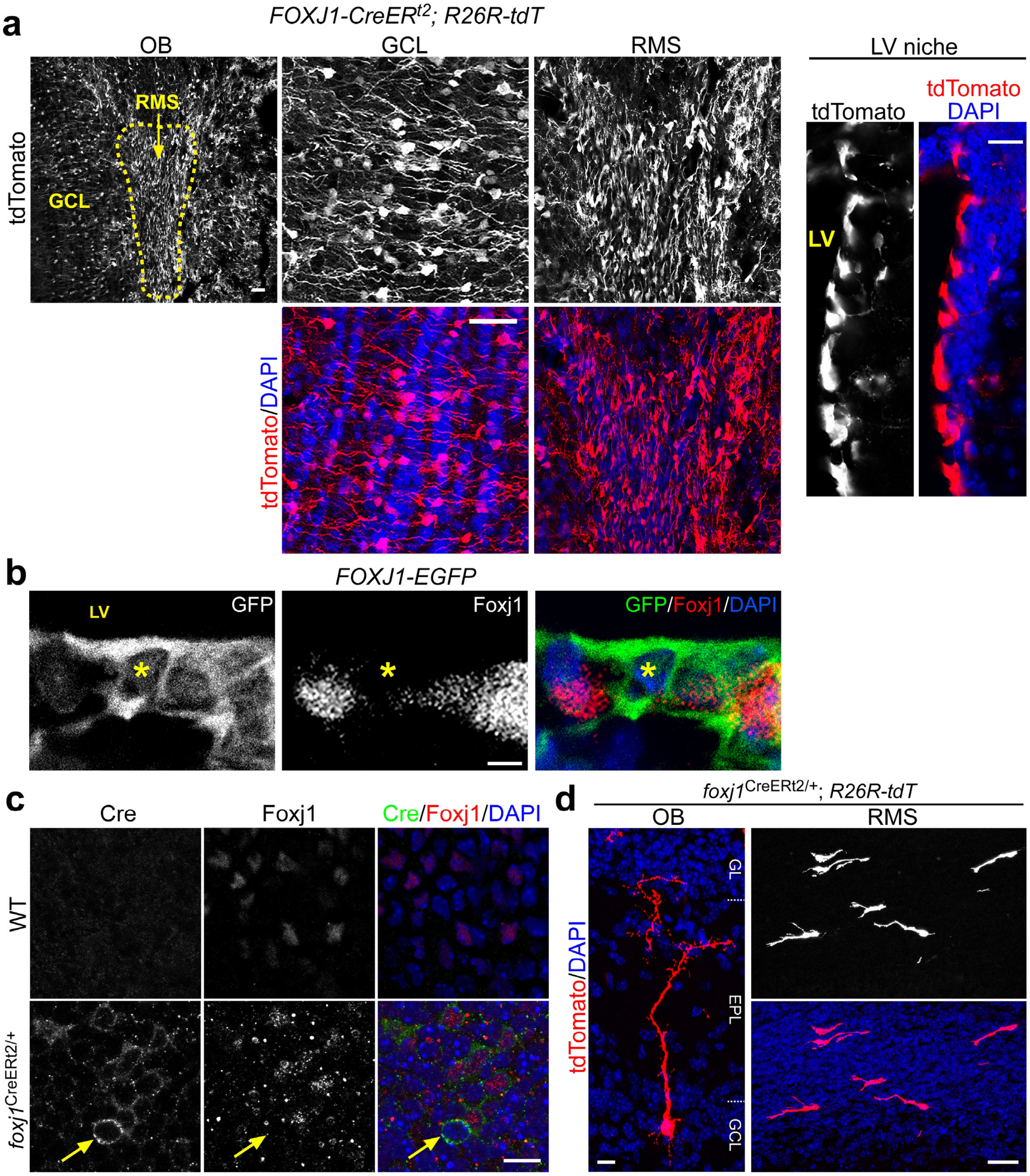

Supplementary Figure 11 (**a**) Representative IHC staining images of olfactory bulb (OB) and corresponding lateral ventricle (LV) niche sections from P28 *FOXJ1-CreER*^{t2}; *R26R-tdT* animal, tamoxifen-induced at P14, stained with RFP antibody and DAPI. Note the extensive tdTomato^{+} cells in the granule cell layer (GCL) and rostral migratory stream (RMS) from *FOXJ1-CreER*^{t2} mediated recombination. Scale bars: 40 μm. (**b**) Confocal images from IHC staining of brain sections from P14 *FOXJ1-GFP* mice (green) for Foxj1 and DAPI, showing GFP^{+} cell lacking Foxj1 expression (*). Scale bar: 5 μm. (**c**) Representative IHC staining of EC wholemounts from P0 wild-type (WT) or *foxj1*$^{CreERt2/+}$ animals, labeled with Cre + Foxj1 antibodies, and DAPI. Arrows indicate an example CreER^{+} cell that is Foxj1-negative. Scale bar: 10 μm. (**d**) Representative IHC staining images from *foxj1*$^{CreER/+}$; *R26R-tdT* animals, tamoxifen-induced at P0, labeled with RFP antibody and DAPI. Left panel: example tdTomato^{+} granule neuron in the olfactory bulb (OB) of P21 animal. GCL: granule cell layer; EPL: external plexiform layer; GL: glomerular layer. Right panel: P10 rostral migratory stream (RMS) showing tdTomato^{+} neuroblasts. Scale bars: 10 μm.

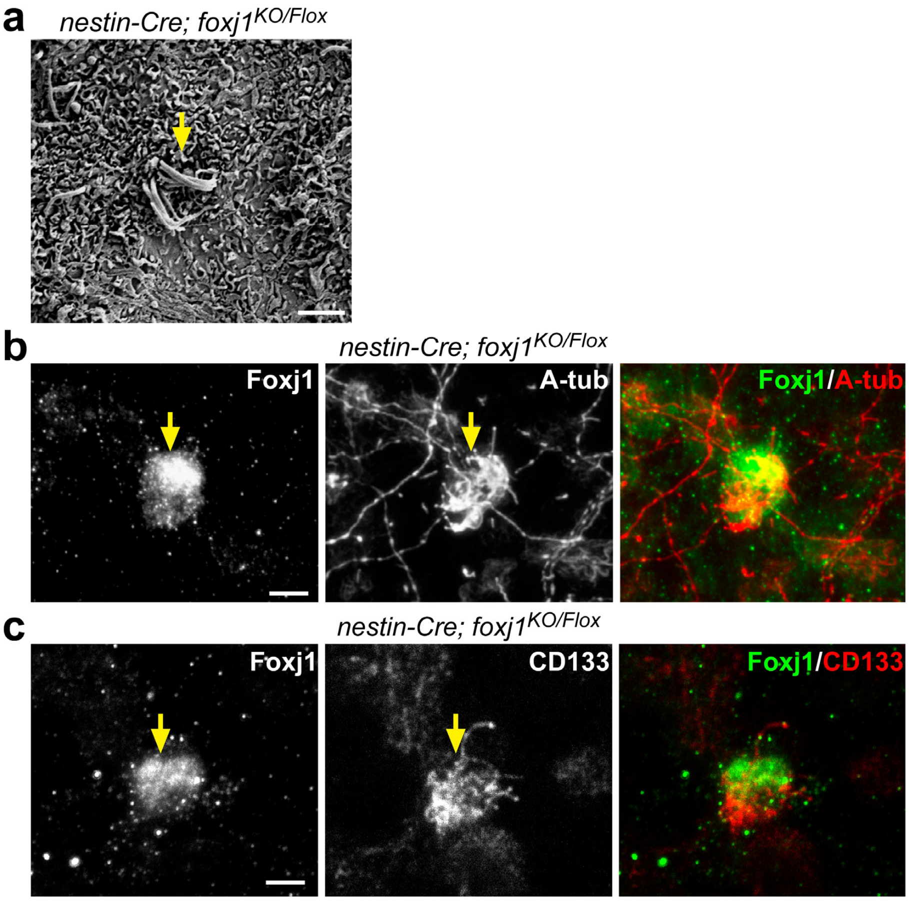

Supplementary Figure 12 (**a**) Scanning electron microscopy image of lateral ventricular surface from P10 *nestin-Cre; foxj1*$^{KO/Flox}$ mutant animal, showing a multiciliated EC (arrow). Scale bar: 5 μm. (**b**,**c**) Confocal images of brain ependymal wholemounts from P10 *nestin-Cre; foxj1*$^{KO/Flox}$ animal, showing IHC staining for Foxj1 + acetylated tubulin (A-tub, **b**), and Foxj1 + CD133 (**c**). Multiciliated cells in mutant animals, identified by either A-tub or CD133 staining of multicilia, were also Foxj1^{+} (arrows). Scale bars: 5 μm.

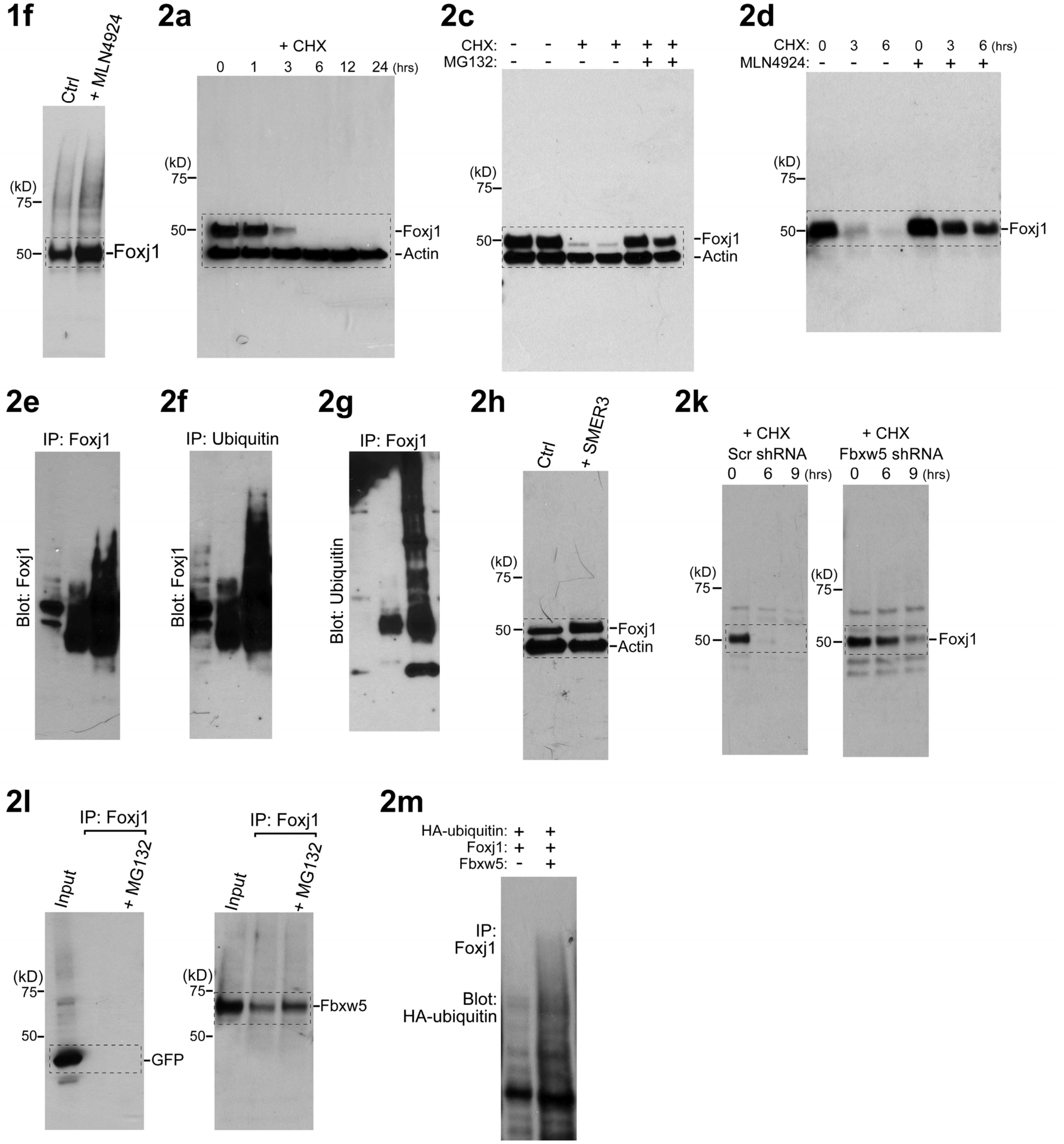

Supplementary Figure 13 Full-length pictures of blots presented in Figures 1 and 2.

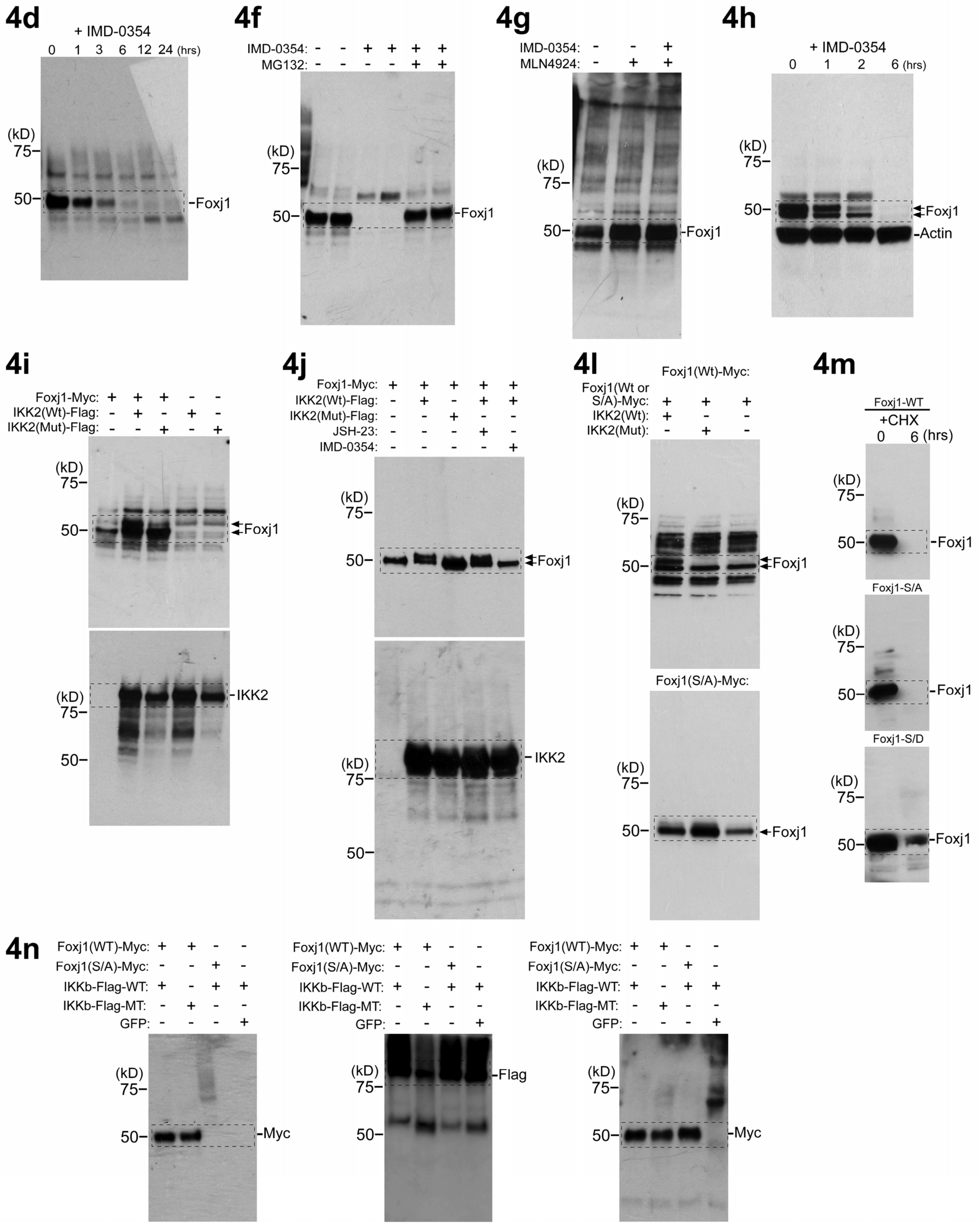

Supplementary Figure 14 Full-length pictures of blots presented in Figure 4.
