# Supplementary material for: Uncovering inherent cellular plasticity of multiciliated ependyma leading to ventricular wall transformation and hydrocephalus
Source: Nat Commun. 2018 Apr 25;9:1655. doi: 10.1038/s41467-018-03812-w (PMC5916891; doi:10.1038/s41467-018-03812-w)
Supplement: Supplementary file 2 — Description of Additional Supplementary Files [file 41467_2018_3812_MOESM2_ESM.pdf]

Description of Additional Supplementary Files

File Name: Supplementary Movie 1
Description: Live-imaging of *foxj1*$^{CreERt2/+}$*; R26R-td* lateral ventricular wall wholemount in low serum condition. This movie shows representative live-imaging of lateral ventricle wholemounts from *foxj1*$^{CreERt2/+}$*; R26R-tdT* mice induced with tamoxifen at P7 and imaged at P14 under low serum (control) culturing conditions. Images were captured continuous once every hour; total time = 60 hours. QuickTime (3.9 MB).

File Name: Supplementary Movie 2
Description: Live-imaging of *foxj1*$^{CreERt2/+}$*; R26R-tdT* lateral ventricular wall wholemount in high serum condition. This movie shows representative live-imaging of lateral ventricle wholemounts from *foxj1*$^{CreERt2/+}$*; R26R-tdT* mice induced with tamoxifen at P7 and imaged at P14 under high serum (progenitor proliferation media) culturing conditions. Images were captured once every hour; total time = 60 hours. QuickTime (4.0 MB).

File Name: Supplementary Movie 3
Description: Live-imaging of *foxj1*$^{CreERt2/+}$*; R26R-tdT* lateral ventricular wall wholemount cultured in high serum condition showing. This movie shows representative tdTomato^{+} cell division (traced by blue dots) in ependymal wholemounts from *foxj1*$^{CreERt2/+}$*; R26R-tdT* mice induced with tamoxifen at P7 and imaged at P14 under high serum (progenitor proliferation media) culturing conditions. Images were captured once every hour; total time = 35 hours. QuickTime (1.6 MB).

File Name: Supplementary Movie 4
Description: Live-imaging of mature primary EC cultures grown in differentiation condition. This movie shows representative lineage-traced tdTomato^{+} mature ECs from *foxj1*$^{CreERt2/+}$*; R26R-tdT* animal imaged under differentiation conditions. Images were captured once every hour; total time = 60 hours. QuickTime (2.8 MB).

File Name: Supplementary Movie 5
Description: Live-imaging of mature primary EC cultures placed in high serum (progenitor proliferation media) condition. This movie shows representative lineage-traced tdTomato^{+} mature ECs from *foxj1*$^{CreERt2/+}$*; R26R-tdT* animal transforming under high serum conditions. Images were captured once every hour; total time = 60 hours. QuickTime (6.6 MB).

File Name: Supplementary Movie 6
Description: Live-imaging of mature primary EC cultures placed in high serum (progenitor proliferation media) condition. This movie shows representative lineage-traced tdTomato^{+} cell division (traced by blue dots) in de-differentiated mature EC cultures from *foxj1*$^{CreERt2/+}$*; R26R-tdT* animal under high serum culturing condition. Images were captured once every hour; total time = 23 hours. QuickTime (2.0 MB).

File Name: Supplementary Movie 7
Description: Live-imaging of *FOXJ1-CreER*^{t2}; *R26R-tdT* lateral ventricular wall wholemount. This movie shows representative live-imaging of lateral ventricle wholemounts from *FOXJ1-CreER*^{t2}*; R26R-tdT* mice induced with tamoxifen at P7 and imaged at P21. Imaged from rostral-dorsal portion of the ventricular wall and the boundary with the rostral migratory stream to capture migrating tdTomato^{+} neuroblasts beneath the ependymal wall surface (visualized by stationary tdTomato^{+} ependymal clones). Images were captured once every 5 minutes; total time = 1.5 hours. QuickTime (10.5 MB).
